# The role of oncolytic virotherapy and viral oncogenes in the cancer stem cells: a review of virus in cancer stem cells

**DOI:** 10.1186/s12935-023-03099-y

**Published:** 2023-10-25

**Authors:** Amirhosein Faghihkhorasani, Alaleh Dalvand, Ehsan Derafsh, Farnaz Tavakoli, Nada Khairi Younis, Saman Yasamineh, Omid Gholizadeh, Pooria Shokri

**Affiliations:** 1 Iran University of Medical Sciences, Tehran, Iran; 2 Tehran Medical Branch, Islamic Azad University of Medical Sciences, Tehran, Iran; 3 Department of Basic Medical Science, Windsor University School of Medicine, Brighton’s Estate, Cayton, St. Kitts And Nevis; 4 Nephrology and Transplantation Ward, Shariati Hospital Tehran University of Medical Sciences, Tehran, Iran; 5 Department of Pharmacy, Al-Noor University College, Nineveh, Iraq; 6 Young Researchers and Elite Club, Tabriz Branch, Islamic Azad University, Tabriz, Iran; 7 Azad Researchers, Viro-Biotech, Tehran, Iran; 8 Department of Medical Science, Faculty of Medical Science, Arak University of Medical Sciences, Arak, Iran

**Keywords:** Viral infection, Cancer stem cell, Oncolytic viruses, Viral oncogenes

## Abstract

Cancer Stem Cells (CSCs) are the main “seeds” for the initiation, growth, metastasis, and recurrence of tumors. According to many studies, several viral infections, including the human papillomaviruses, hepatitis B virus, Epstein–Barr virus, and hepatitis C virus, promote the aggressiveness of cancer by encouraging the development of CSC features. Therefore, a better method for the targeted elimination of CSCs and knowledge of their regulatory mechanisms in human carcinogenesis may lead to the development of a future tool for the management and treatment of cancer. Oncolytic viruses (OVs), which include the herpes virus, adenovirus, vaccinia, and reovirus, are also a new class of cancer therapeutics that have favorable properties such as selective replication in tumor cells, delivery of numerous eukaryotic transgene payloads, induction of immunogenic cell death and promotion of antitumor immunity, as well as a tolerable safety profile that essentially differs from that of other cancer therapeutics. The effects of viral infection on the development of CSCs and the suppression of CSCs by OV therapy were examined in this paper. The purpose of this review is to investigate the dual role of viruses in CSCs (oncolytic virotherapy and viral oncogenes).

**Graphical Abstract:**

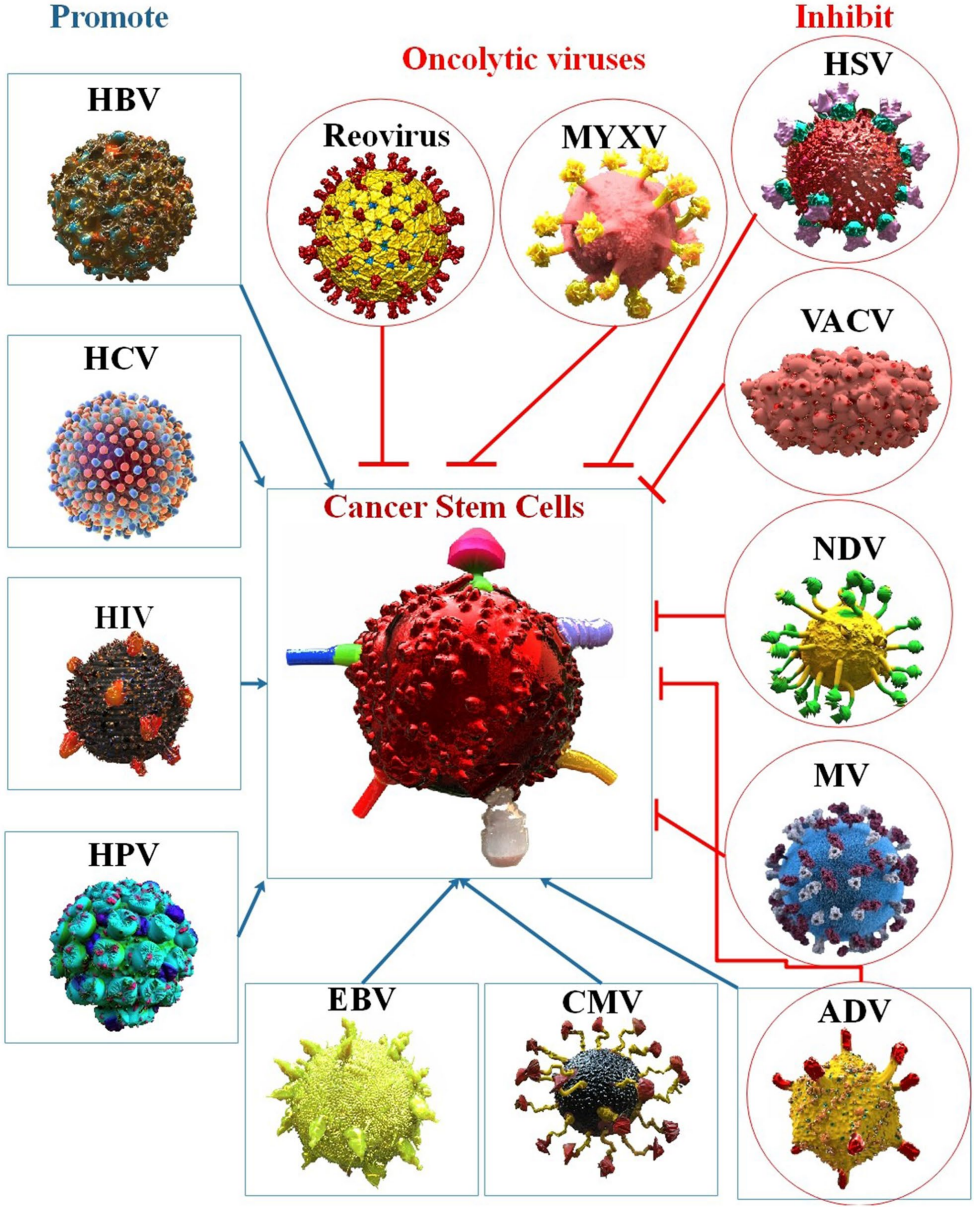

## Introduction

Functional heterogeneity is a result of the distinct driver mutations that exist inside each subclone, which may have a varied effect on the cancer hallmarks. In addition, mounting evidence shows that nongenetic factors, particularly those associated with developmental pathways and epigenetic alterations (such as DNA methylation, histone modification, chromatin openness, microRNA (miRNA), and other noncoding RNA), influence functional heterogeneity. These factors are often attributed to maintaining regular tissue stem cell hierarchies. Similarly, tumor tissues are hierarchically organized by nongenetic factors, and a population of self-renewing CSCs is responsible for the neoplasm’s long-term clonal maintenance [1]. Researchers have spent a lot of time trying to figure out the genetic and epigenetic processes that lead to stemness and CSC formation. Genetic and epigenetic changes would help the CSC group stay alive and add to the ability of tumors to start and grow. Leukemia stem cells showed how DNA methylation is essential for CSC control and tumor growth. Stopping the production of DNA methyltransferase, Dnmt1 stopped leukemia from getting worse. Haploinsufficiency of Dnmt1 also resulted in tumor suppressor gene derepression, fewer bivalent chromatin marks, impaired CSC self-renewal, and delayed leukemogenesis. Even though promoter hypermethylation of a few tumor suppressor genes—which drive oncogenesis at an early stage—was already present in the CSCs and was preserved in the non-stem cancer cells subpopulation, the promoter methylation status of a few CSC markers can reveal differences in both tumor cell populations. Indeed, there are variations in the amount of methylation in the CD133 promoter between CD133+ and CD133 subpopulations isolated from brain and epithelial ovarian cancer [2]. In contrast to healthy cells, CSCs have a markedly changed pattern of gene expression due to an abnormal regulation of the epigenetic machinery. The epigenetic characteristics of CSCs include hypermethylation of CpG islands and inactivation of tumor suppressor genes and/or pro-differentiation proteins. The methylation profile of malignant embryonic stem cells differs significantly from that of CSCs in adult malignancies, it should be noted [3]. The CSC hypothesis was put out 40 years ago and claims that a small number of specialized stem cells drive tumor development like how healthy tissues regenerate [4]. The microenvironment of different tumors has been shown to have distinct areas harboring CSCs; these anatomical areas are known as “niches” much like their healthy counterparts. Numerous studies have so far demonstrated that CSC niches play a role in the upkeep, control of renewal, differentiation, and adaptability of CSCs [5]. Human malignancies such as those of the brain, liver, breast, lung, head and neck, prostate, melanoma, gastric, pancreatic, renal, ovarian, esophageal, and colorectal organs have all been linked to CSC niches [6–8]. In terms of self-renewal and differentiation, CSCs and regular stem cells are identical. Most CSCs are insensitive and resistant to treatments that might result in tumor relapse or recurrence. They also help cancer cells become resistant to chemotherapy and spread, which makes treatments ineffective. Therefore, to eliminate these cells and improve the effectiveness of cancer therapy, improved therapeutic methods are needed [9]. There is an urgent need for a new CSC theory better to explain CSC evolution, biology, and identification and to direct the creation of efficient treatment targets since the processes behind CSC carcinogenesis are still unknown [10].

**Fig. 1:**
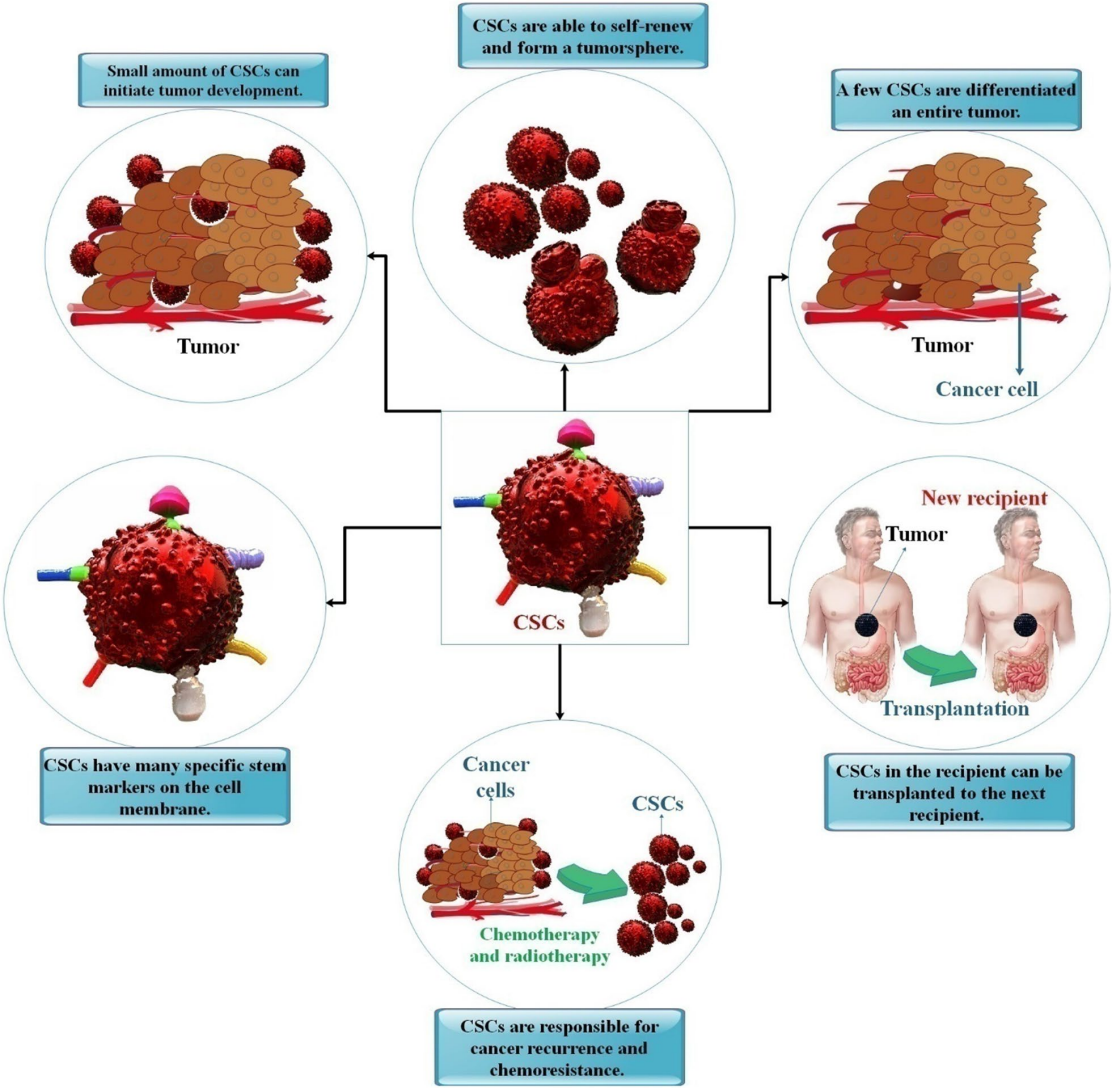
Cancer stem cell (CSC) features. CSCs are identified and assessed as cancer stemness based on five distinct criteria. Self-renewing CSCs may create a tumorsphere. A tumor may be entirely formed by a few CSCs. The recipient’s CSCs may be transferred to the following recipient. Chemoresistance to chemotherapy and tumor recurrence is caused by CSCs. On the cell membrane, CSCs exhibit a variety of distinct stem markers [33]

It is well acknowledged that stem cell markers such as CD24, CD34, CD44, CD133, ALDH1, and ESA is often present in CSCs. It is important to note that distinct tumor types have unique CSC markers for identification and confirmation. For instance, pancreatic CSCs display the cell surface markers CD24, CD44, CD133, ESA, ALDH1, and c-Met [11]. Furthermore, possessing the endless capacity for self-renewal and the ability to start tumors, CSCs also display epithelial-mesenchymal transition (EMT) markers that allow them to migrate and form metastases. EMT plays a significant role in developing tumors, local invasion, metastasis, and treatment resistance. EMT may potentially be related to the growth of cancer cells with stem-like characteristics. Additionally, CSCs have improved drug efflux and DNA repair abilities and overexpression of several detoxifying enzymes, making them resistant to radiation treatment and cytotoxic medications. Therefore, there has been a lot of work done to find new targets and treatments that mainly affect CSCs [12, 13] (Fig. 1).

**Fig. 2:**
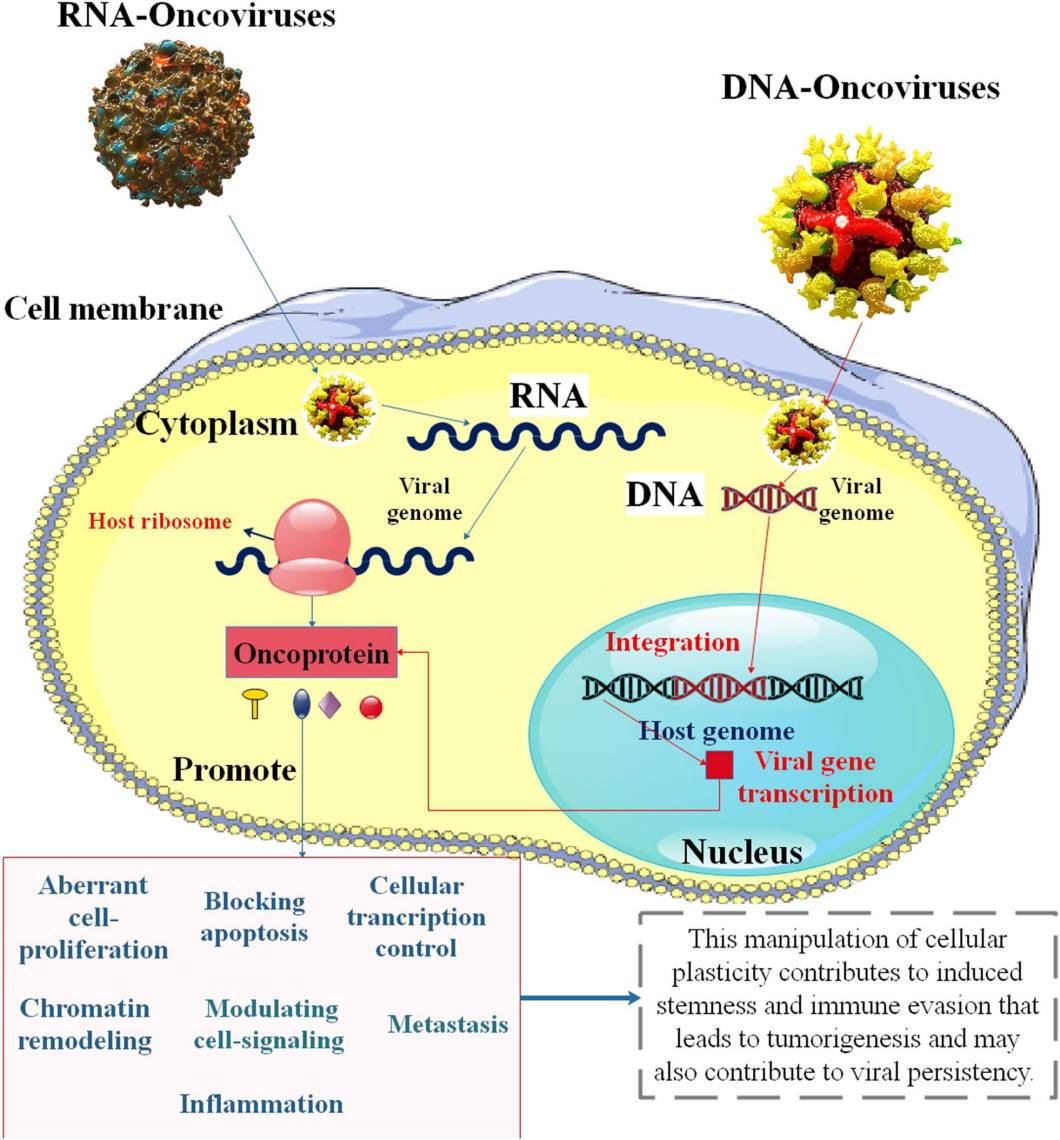
Human tumor viruses can control cellular plasticity to promote stemness, subvert the host immune system, and create long-lasting infections. Different human tumor viruses’ strategies for establishing latency include: While RNA tumor viruses are either transcribed into DNA with subsequent integration into the host genome or stay in the cytoplasm with no integration, DNA tumor viruses either integrate into the host genome or remain episomally, anchoring to the host chromosome. By producing several powerful oncoproteins, oncoviruses cause cellular homeostasis to be dysregulated, which results in the immortalization of the infected cell. Viral oncoproteins influence cell signaling pathways and escape from cellular defense mechanisms, such as inhibiting apoptosis, to promote abnormal cell growth. The subsequent inhibition of cellular metastasis suppressor proteins causes initial malignant cells to spread [34, 35]

Viruses are essential research tools in the study of cancer. In the 1970s, they were utilized to identify the first oncogenes, and they are now being modified to function as anticancer treatments. The capacity of viruses to alter host cell metabolism is essential to both these oncogenic and oncolytic features [14, 15]. The most common cause of mortality globally is cancer, caused by chronic viral infection in 12–15% of cases [16]. According to estimates, several infectious agents, such as the hepatitis C virus (HCV), human papillomavirus (HPV), and Epstein–Barr virus (EBV), may cause cancer in humans [17–19]. Concerning bladder cancer, cholangiocarcinoma, hepatocellular carcinoma (HCC), cervical cancer, nasopharyngeal carcinoma (NPC), gastric cancer (GC), HCV, HPV, and EBV are significant risk factors [20]. Viral infection is an important contributor to cancer, in addition to abnormal DNA methylation or RNA expression. Oncoviruses, or human tumor-associated viruses, are widely cited as the primary initiators of cancer formation. Through disabling the E2F-RB complex, the HPV E7 oncoprotein, for instance, may deactivate the tumor suppressor RB and cause its destruction through the ubiquitin-proteasome pathway [21] (Fig. 2). Evidence of HPV infection demonstrated the virus’ involvement in brain and lung tumors, esophageal cancer, head and neck cancer, cervical cancer, and squamous cell carcinoma of the neck and head [22]. The most prevalent cancers, including pancreatic cancer brought on by chronic pancreatitis (CP), liver cancer brought on by viral hepatitis or alcohol, cervical cancer brought on by the HPV, and GC caused by chronic atrophic gastritis, have been linked to inflammation and immune response. Although there are many potential cancer triggers, the most frequent reason for tumor initiation is chronic inflammation [23]. For example, HCV triggers signaling molecules that encourage the formation of primary hepatocytes and the development of EMT/CSCs [24]. Therefore, the identification of a more effective method for the targeted elimination of CSCs and the knowledge of their regulatory mechanisms in human carcinogenesis may result in the development of a new tool for the management and treatment of cancer [25].

Oncolytic viruses (OV) can infect and replicate in cancer cells. Even non-attenuated viruses that do not infect people are capable of killing cancer cells in a similar manner as attenuated viruses that have been engineered to multiply and lyse cancer cells while sparing healthy tissues. The therapeutic efficacy of more significant viruses may also be increased by equipping them with immune-stimulating transgenes [26]. Without genetic editing, OVs can kill cancer cells, but since they lack selectivity, they may also harm healthy cells. Numerous preclinical studies have modified and investigated various OVs, some of which have led to clinical trials in cancer patients [27]. Due to its multiple anti-cancer pathways, oncolytic virotherapy has become a topic of increasing interest in cancer research. For instance, OVs may directly lyse both highly proliferative cells (non-CSCs) and quiescent (CSCs) cells, unlike chemotherapeutic medicines pushed out of infected cells via ABCG2. Other strategies include the formation of anti-tumor immunity and indirect death of cancer cells that are not infected, such as the destruction of tumor vasculature. CSCs are the root of tumor recurrence and treatment resistance. However, OVs have been shown to have particular anti-tumor effects against these cells [28, 29]. On the whole, it has been demonstrated that OVs have remarkable potential for identifying CSCs and preventing tumor recurrence. This method of tumor eradication departs from standard methods and provides a unique means of extending therapeutic effectiveness to drug-resistant CSCs. As scientists continue to clarify the specific mechanisms behind CSC persistence, tumorigenicity, and resistance, new viruses may be developed to limit CSC activity further and, hence, improve outcomes [30–32]. In this research, we looked into how viral infection influences CSC formation and how oncolytic virus treatment may decrease CSC. The purpose of this review is to investigate the dual role of viruses in CSCs (oncolytic virotherapy and viral oncogenes). This study helps the researchers about the importance of the role of viruses in CSCs.

## Viral infection promotes cancer stem cells in different cancers

Oncovirus infections are widespread and sporadically lead to cancer. Generally, all oncoviruses promote tumorigenesis utilizing metabolic pathways. The method by which oncoviruses cause cancer varies by the oncovirus genetic material DNA, RNA, and retroviruses. The major oncoviruses are Epstein–Barr virus, hepatitis B virus (HBV), HCV, mastadenovirus, aviadenovirus, rhadinovirus, varicellovirus, polyomavirus, adenoviruses, orthopoxvirus, leporipoxvirus, parvovirus, human immunodeficiency virus (HIV), HPV, human herpesvirus 8, and simplex virus which induce uncontrolled cell growth by a common way of interfering with the tumor p53 gene [32, 36, 37]. In addition, according to many studies, some oncoviruses, including the HPVs, HBV, Epstein–Barr virus, and HCV, promote the aggressiveness of cancer by encouraging the development of CSCs features [20, 38, 39]. In the following section, we have shown the role of different types of viruses in CSCs.

### Papilloma virus and cancer stem cells

**Fig. 3:**
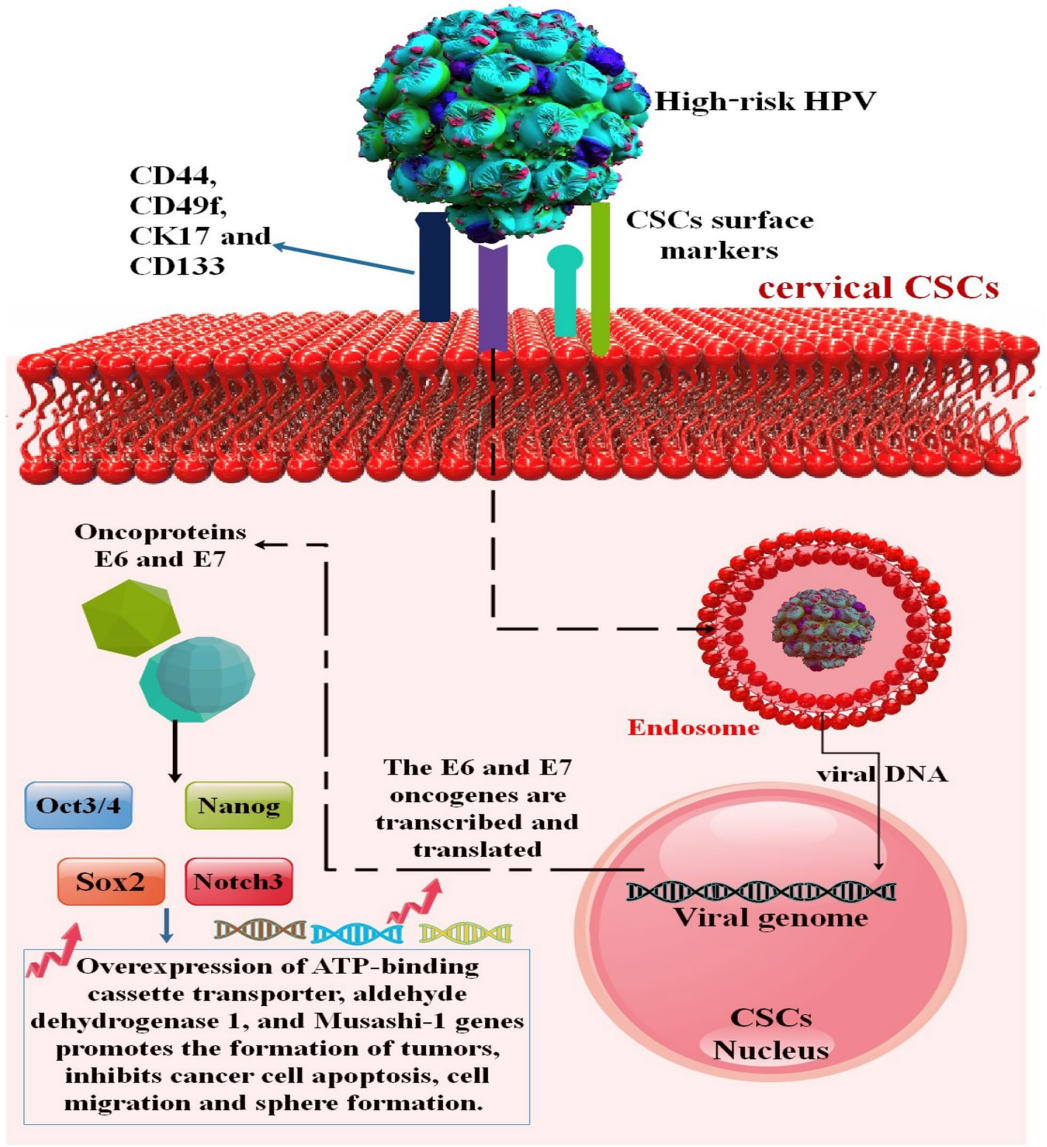
Cervical CSC network modulation by high-risk HPV oncoprotein. With the help of the surface markers CD44, CD49f, CK17, and CD133, high-risk HPV targets explicitly stem cells in the cervical epithelium. High risk-HPV attaches to cell surface receptors, internalizes, and transports viral DNA to the cell nucleus. The oncoproteins E6 and E7 are produced due to the transcription and translation of the E6 and E7 oncogenes. The stemness-related genes Oct3/4, Nanog, Sox2, and Notch3 are among the molecules that E6 and E7 target; their overexpression encourages the growth of tumors and prevents the apoptosis, cell migration, and sphere formation of cancer cells. By upregulating ATP-binding cassette transporter, aldehyde dehydrogenase 1, and Musashi-1 expression, Oct3/4, Nanog, Sox2, and Notch3 promote drug resistance, which in turn encourages the clonogenicity, proliferation, increased invasiveness, and chemoresistance of cervical CSCs [52]

This virus is highly tropistic for the epithelia lining the nasal cavity, genitalia, and skin. Based on their propensity to cause cancer, HPV is classified as either low-risk (causing mostly non-cancerous diseases) or high-risk (causing deadly diseases) [40]. HPV infection is the main reason why women get cervical cancer. Genomic instability may emerge from the integration of the virus genome into the host chromosome, as is the case with the oncogenes HPV E6 and E7, which are encoded in the early € region of the HPV gene [41, 42] (Fig. 3). Additionally, oropharyngeal squamous cell carcinoma (OPSCC) with HPV is distinct from OPSCCs without HPV in terms of epidemiology, clinical features, and molecular traits. OPSCCs with HPV are primarily OPSCCs [43]. Unlike the more prevalent vaginal malignancies, esophageal squamous cell carcinoma (ESCC), colorectal cancers, conjunctival carcinomas, and oropharyngeal cancers, it is believed that HPV contributes to the development of neoplastic changes within the stomach mucosa, which may lead to GC [40, 44].

Researchers found that cervical CSC-like cells are favorable for the HPV receptors CD49f and AII a study, indicating that cervical stem cells may have been infected with the virus and may be to blame for developing cervical cancer and supporting tumor growth [45]. As its viral DNA is integrated into the genome of basal cells with stem cell-like characteristics, HPV has a greater chance of causing an oncogenic transformation into cervical CSCs. The transformation zone (TZ) or squamocolumnar junction, where the ectocervix and endocervix converge, is where the infection manifests itself. The TZ has a distinct shape and gene expression profile, expressing genes such as matrix metalloproteinase-7, anterior gradient 2, CD63, and keratin 7. The EMT is another critical element in the development of cervical cancer. EMT is an essential step for the development of invasive cells and metastasis, and it is controlled by the transcription factors twist family bHLH transcription factor 1 (TWIST1) and snail family transcriptional repressors 1 (SNAI1) and 2 (SNAI2). The stimulation of the EMT enhances metastasis, tumor cell invasion, and treatment resistance since it is a rich source of CSCs [46, 47].

The self-renewal associated transcription factor Sox2 was upregulated in HPV-related head and neck squamous cell cancer (HNSCC) due to the activation of the PI3K-AKT pathway by HPV E6/7. Functional characteristics of the HPV-associated HNSCC side population, including CSC markers, CD44, CD24, and ALHD1, are maintained in the final product [48]. The frequency of CSCs in HPV(+) vs. HPV(−) HNSCC cell lines is not significantly different. Differential cisplatin resistance between HPV(+) and HPV(−) cells in HNSCC is seen only in CSC. After being sorted, HPV(−) cancer cells did not form many colonies. Colony formation was up in both CSC and non-CSC after transduction with HPV E6/E7. The presence of CSC in HNSCC is correlated with HPV status. The clonogenicity of HPV(+) cells and HPV(+) cells transduced with HPV E6/E7 is higher than that of HPV(-) cells. CSC in HNSCC is resistant to cisplatin treatment. This indicates that conventional chemotherapeutic medicines may reduce tumor size by targeting and killing off non-CSC, but CSC has mechanisms that allow them to avoid death. CSC response to cisplatin treatment is independent of HPV status, therefore, the superior results seen in HPV(+) cancer patients likely result from other circumstances [49]. In another study, investigators evaluate HPV statuses and the dynamic responses in populations with a CSC phenotype in HNC cell lines after exposure to X-rays at therapeutic doses. There was no association between differences in CSC density between HPV groups and the improved clinical outcomes seen in the HPV-positive status. The CSC population was 1.9–4.8% in HPV-positive cell lines and 2.6–9.9% in HPV-negative cell lines. However, HPV-negative cell lines showed significantly higher CSC proportional increase following very-low-dose radiation (4 Gy) X-irradiation, being 3-fold that of the HPV-positive group at 72 h post-irradiation. Radiation therapy at therapeutic doses may alter the percentage of tumor cells that are CSCs. These results highlight that clonogenic treatment response may be more illuminating of therapy outcomes than inherent population density alone, implying a possible effect of etiology on radio-responsiveness in CSCs [50].

Overall, HPV and CSCs have not yet lived up to their potential as biomarkers of treatment response. Learning how HPV-positive and -negative aetiologies affect CSC response to treatment and tumor plasticity will allow for more precise targeting of treatments [51].

### Viral hepatitis and cancer stem cells

A viral infection is the root cause of viral hepatitis, an inflammatory liver disorder. Although other viruses can occasionally inflame the liver, hepatitis virus infections are the most common cause of viral hepatitis. Chronic viral hepatitis B or C infection is a significant risk factor for progressing to HCC. In reality, the global distribution of HCC is linked to the prevalence of either the HBV) or the HCV. Hepatitis virus pathogenesis often includes a series of adverse events. Beginning with a cellular immune response, it progresses to liver fibrosis, cirrhosis, and ultimately HCC by causing DNA damage, mitochondrial malfunction, and endoplasmic reticulum stress [17, 18, 53].

In chronic hepatitis, liver cirrhosis, and HCC, CSC clusters with CD133+ and/or CD44+ characteristics have been found. Significant correlations were found between the expressions of CD133 and CD44 and more outstanding tumor grades, fibrosis stages, and inflammatory activity. To develop new treatments for HCC targeting and prevention, it may be helpful to evaluate CD44 and CD133 expression patterns as CSC markers in non-neoplastic liver and HCCs [54]. A subset of tumor cells known as liver CSCs (LCSCs) is capable of causing the onset and recurrence of cancer. In addition, in an investigation, a control group, six groups of patients with HCV, HCV + cirrhosis, HCV + HCC, HBV, HBV + cirrhosis, or HBV + HCC were examined for LCSC levels, and miR-1290 and miR-1825 expression. In the etiology of liver cirrhosis and HCC, CD133/EpCAM-expressing cells emerged due to either chronic HCV or HBV infection. In comparison to the control group, the percentages of LCSCs that express CD133/EpCAM were higher in viral hepatitis and cirrhosis groups. The proportion of LCSCs was more increased in HCC patients. Significant relationships between stemness-associated microRNAs, miR-1290 and, miR-1825, and CD133/EpCAM-expressing cells were observed. Additionally, miR-1290 and miR-1825 levels were significantly elevated in the cirrhosis and HCC groups associated with viral hepatitis. Tumor size and number were also positively correlated with the expression of miR-1290 and miR-1825 in HCV + HCC. Only miR-1825, however, could differentiate between HCC subtypes linked with HCV and HBV. MiR-1290, miR-1825, and LCSCs that express CD133/EpCAM have the best sensitivity and specificity for identifying HCC [25].

#### HBV and cancer stem cells

More and more evidence points to the existence of hepatic CSCs (hCSCs), which have a role in chemotherapy resistance and cancer recurrence after treatment or surgery. Epigenetically, downregulation of the chromatin-modifying Polycomb Repressive Complex 2 (PRC2) during HBV infection leads to re-expression of hCSC marker genes in infected hepatocytes and HBV-associated liver tumors. On the other hand, besides the expression of hCSC markers, the formation of hCSCs requires cellular changes, metabolic rewiring, cell survival, evasion of programmed cell death, and immune evasion [38].

The expression of CSC-related genes (Klf4, Sox2, Nanog, c-Myc, and Oct4) and markers (CD133, CD117, and CD90) is also upregulated by HBV in human hepatoma cells, as is CSC self-renewal. Human hepatoma cell and clinical cancer tissue studies have shown that HBV enhances CD133 and CD117 expression in HCC tissues and promotes the formation of CSCs. It also fosters hepatoma cell growth and migration. In-depth research has shown that the HBV PreS1 protein is necessary for developing CSCs mediated by the virus. PreS1 increases the capacity of normal hepatocyte-derived cell line (L02) and human hepatoma cell line (HepG2 and Huh-7) to induce tumorigeneses in nude mice by activating CD133, CD117, and CD90 expression in both cell lines. It also promotes L02 cell migration, growth, and sphere formation. As a result, PreS1 functions as a novel oncoprotein and is crucial for the emergence and self-renewal of CSCs throughout the development of HCC [55].

**Fig. 4:**
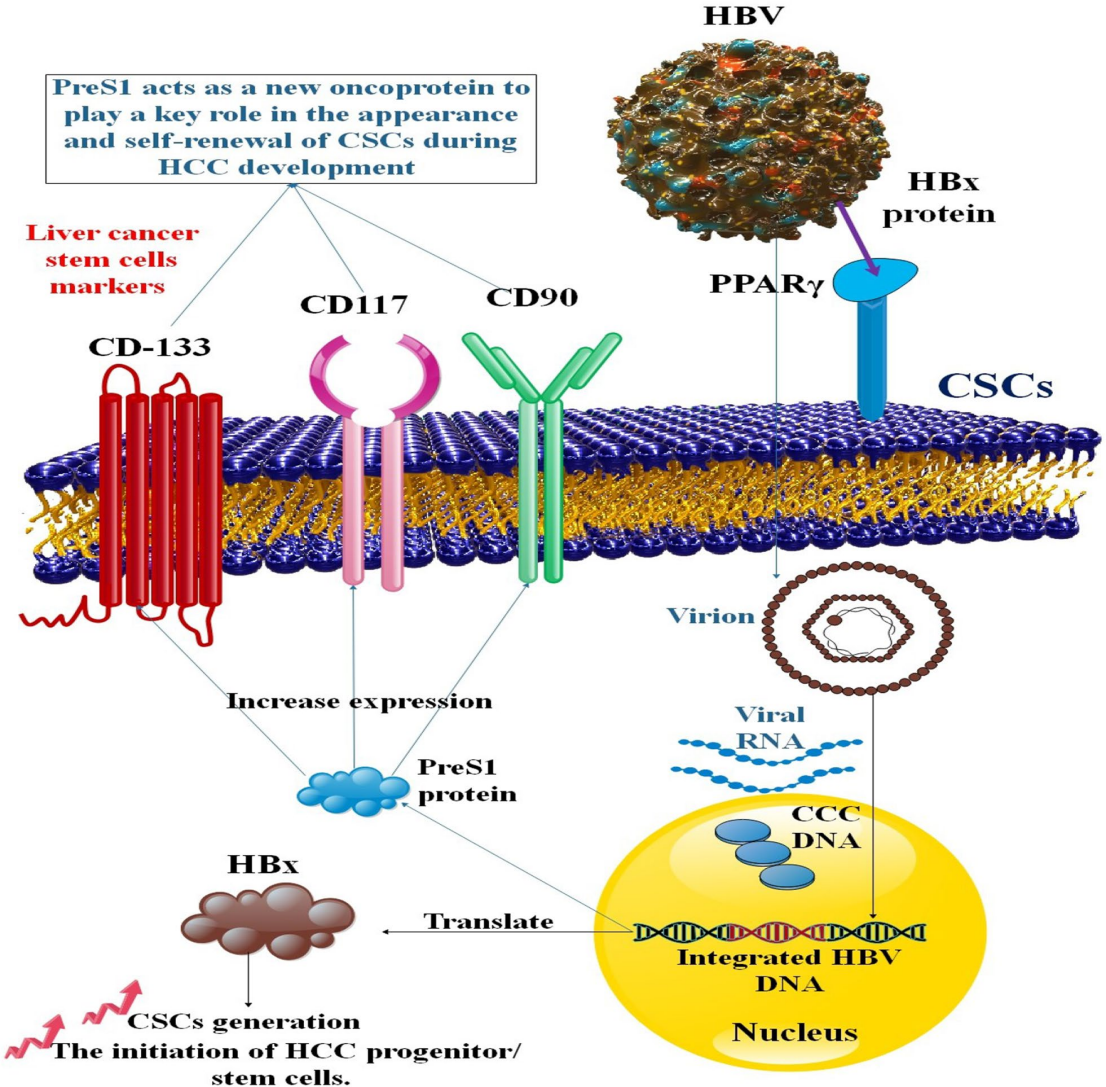
CSC production was stimulated by HBV. HBx promotes AFP synthesis to stimulate the normal reprogramming of liver cells, which is essential for developing HCC progenitor/stem cells. Among the several cell lines that originate from normal hepatocytes, PreS1 upregulates the expression of CD133, CD117, and CD90. PreS1 acts as a novel oncoprotein, vital in the establishment and maintenance of CSCs during HCC’s pathogenesis

The HBV-X protein (HBx) is essential for causing the liver cells to convert into cancerous tissue. HCC is influenced by HBx and pathways connected to stem cell self-renewal. By encouraging gene expression changes indicative of CSCs, HBx contributes to the development of hepatocarcinogenesis, at least in part [56]. Hepatocarcinogenesis is also connected to the expression of alpha-fetoprotein (AFP). HBx promotes AFP synthesis to stimulate the normal reprogramming of liver cells, which is essential for developing HCC progenitor/stem cells. AFP may be a novel biotarget for inhibiting HBV-induced hepatocarcinogenesis [57]. Through PI3K/Akt signaling, miR-124 overexpression or lncRNA-MALAT1 silencing prevented HBx-induced CSC generation, stemness-related factor activation, and tumorigenicity [58] (Fig. 4).

#### HCV and cancer stem cells

The differentiation state and motility of HCV-induced CSCs are critical features in the progression of severe liver disease [59]. CSC characteristics are acquired when an HCV sub-genomic replicon is expressed in cultured cells. These characteristics include increased expression of Lgr5, CD133, AFP, cytokeratin-19 (CK19), Lin28, and c-Myc, as well as doublecortin and CaM kinase-like-1 (DCAMKL-1), CK19, and Lgr5. On the other hand, the expression of these factors is reduced when the replicon is removed from these cells. When a group of pluripotency factors is overexpressed, the putative stem cell marker DCAMKL-1 is likewise increased. Fluorescence-activated cell sorting (FACS) is used to extract DCAMKL-1-positive cells from hepatoma cell lines, and these cells grow into spheroids in Matrigel. The reduction of DCAMKL-1 caused by small interfering RNA (siRNA) greatly lowers the levels of NS5B and HCV RNA. High levels of DCAMKL-1, CK19, AFP, and active c-Src expression/co-expression are indicative of this HCV-induced phenotype. Through the induction of DCAMKL-1 and hepatic progenitor and stem cell-related factors, chronic HCV infection appears to predispose cells towards the path of acquiring CSC-like traits [60].

In a variety of organs, osteopontin (OPN) contributes to cell migration, proliferation, and tumor growth. OPN interacts with CD44 to promote stemness. However, it is yet unknown how HCV replication and OPN-mediated IFN signaling in stem cell populations work. Additionally, OPN markedly reduces the expression of genes induced by IFN and significantly increases HCV replication in EpCAM+/CD44+ CSCs. The epithelial cell adhesion molecule (EpCAM)+/CD44+ CSC population and OPN expression are both increased by the GSK-3β inhibitor BIO, which also inhibits IFN signaling via degrading STAT1. OPN increases HCV replication in EpCAM+/CD44+ CSCs while simultaneously inhibiting STAT1 phosphorylation and degradation, which hurts the IFN signaling pathway [61]. In addition, plasminogen activator inhibitor-1 (PAI-1)-mediated AKT activation in hepatocytes caused by HCV infection promotes the CSC state. As a result, altering PAI-1 activity may provide new therapies to stop the onset of HCV-related chronic liver disorders [62]. In another study, HCV-infected hepatocytes show sphere formation on very low-adhesion plates with typical activation of the CSC signaling pathway in NOD-SCID IL2R gamma null (NSG) mice. There was a more significant impact from sorafenib and static therapy that resulted in HCV-associated CSC mortality [24].

### Epstein–Barr virus and cancer stem cells

Latent membrane protein 1 (LMP1), the main oncoprotein of the EBV, is linked to human malignancies, particularly NPC, and it promotes tumor cell invasion, metastasis, and EMT. LMP1 may alter epithelial cells’ phenotype such that it resembles cancer progenitor cells (CPC) rather than CSC, and this suggests that LMP1-induced phenotypic alterations aid in the formation of NPC [63]. LMP1 also allows NPC to develop cancer stem cell traits by activating the mTORC1 and mTORC2 pathways. Different tumorigenic characteristics are caused by various mTOR pathways. For CSC drug resistance, mTORC1 must be insensitive to rapamycin. The primary cause of NPC tumor initiation is mTORC2 signaling. The migration and invasion of NPC cells are controlled by mTORC1 and mTORC2. Identifying mTOR signaling’s functions in the development of NPC CSCs has implications for innovative treatment approaches to treat relapsed and metastatic NPC and achieve long-lasting remission [64].

The monoclonal proliferation of EBV-infected epithelial cells characterizes EBV-associated gastric carcinoma (EBVaGC), one of four molecular sub-types of GC that together account for 10% of all gastric carcinomas. An inquiry of CSCs in EBVaGC requires two methodologies since EBVaGC comprises EBV-infected epithelial cells. Finding cellular elements that contribute to CSC proliferation, such as crucial signaling pathways, is one strategy. NPC is another EBV-associated epithelial neoplasm that benefits from CSC maintenance through the NF-κB, Notch, PI3K/AKT, Hedgehog, and Wnt/β-catenin pathways. Finding viral components that may be important for CSC proliferation is the second strategy. The expression of EBV-determined nuclear antigen 1 and LMP2A is observed in EBVaGC, representing a subset of EBV-latent proteins that are present in these cells. The latter is crucial in transforming cancer cells, altering cell motility, preventing cell differentiation, and promoting anchorage-independent cell proliferation. Although LMP1 is hardly ever expressed in EBVaGC, LMP2A also aids in developing CSC in NPC. Furthermore, it was observed that the subset of cancer cells expressing CD44v6/v9+/+ exhibited a greater abundance of characteristics associated with CSCs, thereby suggesting that CD44v6/v9 serves as a distinctive CSC marker for EBVaGC. The LMP2A-NFB route was used to stimulate the capacity to form spheres, and EBVaGC may be treated by targeting this pathway [65].

### Cytomegalovirus and cancer stem cells

Recent research on the human cytomegalovirus (HCMV) has focused on how it affects the growth of cancer and its well-known effects in immunocompromised people. In cancer, HCMV may facilitate onco-modulation, a mechanism that speeds up the development and dissemination of the tumor. While the onco-modulation paradigm has effectively treated certain tumors infected with HCMV, it is insufficient in accounting for all of the observed biological phenomena in these tumors. The collective evidence regarding the pro-oncogenic potential of HCMV proteins, activation of pro-oncogenic pathways in HCMV-infected cells, in vitro transformation of HCMV-infected cells, sustained growth of CTH cells exhibiting an HCMV signature, tumorigenicity of CTH cells upon injection in NSG mice, and compliance with the WHO classification system criteria, all support the inclusion of HCMV in the roster of human oncoviruses [66]. The onset of HCMV infection in individuals with a sound health status is characterized by viremia and the lytic cycle, which is subsequently succeeded by latency. Immunosuppressive states, acute bacterial and viral infections, transplantation, and physiological immune suppression during pregnancy can disturb the established homeostatic balance between the host and HCMV. In reality, during a lifetime, HCMV will genetically and phenotypically change to adapt to a changing immunological environment. In certain situations, this evolution might result in the emergence of CSCs, which would then result in the formation of “oncogenic” strains [67]. Despite aggressive surgical resection, chemotherapy, and radiation treatment, glioblastoma (GBM) has an inferior prognosis. Unfortunately, glioma CSCs (GCSCs), a subpopulation of GBM cells that can cause recurrent tumors, are not targeted by this standard therapy. The degree of HCMV immediate-early (IE) protein expression in GBMs has been a predictive indicator of poor patient survival. GBMs express HCMV proteins. In vitro testing was done to determine the degree to which HCMV infection of primary GBM cells resulted in GCSC phenotype. A significant portion of CD133-positive cells in initial GBMs expressed HCMV-IE, and greater co-expression of these two proteins was associated with a worse chance of patient survival. When GBM cells were infected with HCMV, CD133 and other GSCS markers (Notch1, Sox2, Oct4, Nestin) were upregulated. Additionally, HCMV infection promoted the development of GBM cells as neurospheres, a characteristic of GCSCs. This phenotype was inhibited by either chemical inhibition of the Notch1 pathway or by administration of the antiviral medication ganciclovir. HCMV-IE-expressing GBM cells were unable to develop into neuronal or astrocytic phenotypes. The phenotypic flexibility that HCMV infection causes in GBM cells to boost GCSC characteristics may increase the tumor’s aggressiveness [68]. Additionally, HCMV might promote stem cell survival, which might support the development of cancer. The IE1 protein has been observed to enhance the expression of essential stemness markers such as SRY-Box Transcription Factor 2 (SOX2), Nanog, Nestin, and octamer-binding transcription factor 4 (OCT3/4), thereby promoting the proliferation of GBM-CSCs. Promoting transcription factor expression in GBM cells by the HCMV IE1 protein is crucial for the survival of CSCs, tumor development, and signaling pathways associated with the EMT phenotype [68–70]. In contrast to HCMV-negative tumor-associated macrophages (TAMs), the secretory profile of HCMV-positive TAMs exhibited increased levels of the IL-6, IL-8, and monocyte chemoattractant protein-1 (MCP-1). Furthermore, compared to control cells, the secretome of TAMs infected with HCMV elicited the upregulation of genes associated with Breast Cancer Stem Cells (BCSC), colony formation, invasion, and proliferation in SUM149 cells. Moreover, the phosphorylation of intracellular signaling molecules such as p-STAT3, p-AMPKα, p-PRAS40, and p-SAPK/JNK was induced in SUM149 cells upon exposure to the secretome of HCMV-positive tumor-associated macrophages. The findings of this study indicate that the secretion profile of TAMs infected with HCMV leads to heightened proliferative, invasive, colony-forming, and BCSC traits through the phosphorylation of intracellular signaling molecules such as p-STAT3, p-AMPKα, p-PRAS40, and p-SAPK/JNK in cells of inflammatory breast cancer [71].

### HIV and cancer stem cells

HIV proteins, including the envelope protein gp120, accessory protein negative factor Nef, matrix protein p17, transactivator of transcription Tat, and reverse transcriptase RT, are intrinsically carcinogenic, cause oxidative stress, and be released from cells that are infected with or producing them. These characteristics are thought to be the basis for their ability to influence unrelated epithelial cells, resulting in their malignant transformation and increasing the tumor-genic potential of already transformed/cancer cells. HIV proteins can act alone or in concert with other known oncoproteins to cause most cancers in HIV-1-positive people taking antiretroviral therapy. These oncoproteins originate from human hepatitis B and C viruses and HPVs with a high carcinogenic risk [72]. It has been hypothesized that memory T lymphocytes may possess a limited population of stem cell-like cells, similar to several organs where mature, terminally differentiated cells are supplied by long-lasting stem cells of lesser differentiation. Nevertheless, the identification and isolation of these cells from humans, mice, and nonhuman primates have only been achieved recently. These cells, known as “T memory stem cells” (TSCM), make up about 2–4% of all circulating T lymphocytes, appear to be very resilient, and have the ability to quickly differentiate into more mature central memory, effector memory, and effector T cells while keeping the size of their pool through homeostatic self-renewal. According to recent research, CD4+ TSCM cells are a key component of the HIV-1 reservoir in people on suppressive antiretroviral treatment (ART), and their relative resistance to SIV is an important characteristic of non-pathogenic SIV infection in natural hosts [73]. The predominant composition of the HIV-1 reservoir in individuals undergoing suppressive ART is constituted by CD4+ T memory stem cells (TSCM). In natural hosts, non-pathogenic HIV infection is characterized by the CD4+ TSCM cells’ relative resistance to HIV. Furthermore, the persistence and regulation of HIV-1 latency within newly identified CD4+ TSCM, which possess self-renewal and differentiation capacities that bear a resemblance to undifferentiated CSCs, imply that comparable intracellular signaling pathways and cellular effectors may typify quiescence or latency in both CSCs and dormant HIV-1 reservoirs, and could be leveraged to impede self-renewal and elicit “activation”, differentiation, and/or apoptosis in both [73]. CSCs are a subset of cancer cells that share traits with ESCs, such as self-renewal, multi-lineage differentiation, and the capacity to start carcinogenesis. CSCs are generally resistant to chemoradiation treatment. It’s interesting to note that intracellular processes that support quiescence and encourage self-renewal in adult stem cells (ASCs) and CSCs also probably help keep HIV-1 latent in CD4 + memory T cells. The development of substances that can selectively reactivate the latent virus, also known as the “shock and kill” approach, is required due to the persistence of latent but replication-competent proviruses despite antiretroviral therapy’s high effectiveness in controlling HIV-1 replication. In the absence of T cell activation and differentiation, it has been demonstrated that homeostatic proliferation in central CD4+ memory T (TCM) cells, a memory T cell subset that exhibits limited self-renewal and differentiation and is a primary reservoir for latent HIV-1, strengthens and stabilizes the latent reservoir. In a newly identified subgroup of CD4+ T cells called TSCM cells, HIV-1 has also been reported to establish persistent and long-lasting latency. TSCM cells, as opposed to TCM cells, show stem cell characteristics, such as self-renewal and differentiation into all memory T cell subsets, that are more akin to ESCs and ASCs. The hypothesis put forth by researchers suggests that the activation of AMPK, a key regulator of cellular metabolism involved in T cell activation and the differentiation of ESCs and ASCs, may result in the reactivation of latent HIV-1 during T cell activation. This reactivation could potentially aid in the destruction of the virus. Additionally, the activation of AMPK may induce the differentiation, activation, or apoptosis of CSCs, thereby inhibiting the development of tumors. The researchers further put up the innovative proposition that drugs that have shown the ability to assist the reactivation of latent HIV-1 and promote the differentiation and death of CSCs (such as bryostatin-1, JQ1, metformin, butyrate, among others) are likely to achieve these effects by activating AMPK via a shared mechanism [74]. There is currently a lack of comprehensive and empirical research examining the precise impact of HIV on the formation and proliferation of CSCs across various cancer types. By doing more investigation in this domain, we may contribute to the advancement of diverse therapeutic approaches for managing malignancies associated with HIV.

### Adenovirus and cancer stem cells

Over time, adenovirus (ADV) has been tailored to meet the requirements of oncolytic virotherapy for cancer and human gene therapy. While specific viral components may elicit innate immune responses, ADV is generally considered a safe option for gene therapies due to its infrequent integration into the host genome [75, 76]. ADV infection of patient-derived glioma cells speeds up the formation of tumorspheres. Tumorspheres infected with ADV demonstrated the ability to undergo self-renewal and differentiation into multiple cell lineages through the upregulation of stem cell markers. The likelihood of xenograft tumor development was higher in tumorspheres infected with ADV in mice with compromised immune systems. The upregulation of TLR9 after ADV infection and the observed reduction in ADV-induced GSCs upon TLR9 knockdown suggest a plausible association between ADV infection and GSC formation through TLR9. The results indicate that ADV-induced GSCs exhibit increased expression of MYD88, total STAT3, and phosphorylated STAT3. The stemness of ADV-induced GSCs was observed to diminish upon the knockdown of MYD88 or the pharmaceutical inhibition of STAT3. The study’s findings indicate that ADV infection resulted in an elevation of long non-coding RNA NEAT1. The knockdown of NEAT1 resulted in a decrease in the stemness of GSCs that were generated through the use of ADV. Finally, it is noteworthy that glioma cells exhibited an increase in stemness markers upon exposure to HMGB1, a type of damage-associated molecular pattern (DAMP) that triggers TLR signaling [77] (Table 1).

**Table 1:** The role of viral infection in the enhancement of CSCs

Viral infection	Cancer type	Effects on CSCs	Ref.
HPV	Head and neck squamous cell carcinoma	There is a correlation between HPV status and the percentage of CSC in HNSCC. The fact that CSC response to cisplatin treatment is unaffected by HPV status raises the possibility that additional variables account for the superior prognosis for people with HPV(+) cancer	[49]
HPV	Head and neck squamous cell carcinoma	No link was seen between differences in CSC density between HPV groups and the improved clinical results observed in the HPV-positive status	[50]
HCV and HBV	Liver cancer	In comparison to the control group, the percentages of LCSCs that express CD133/EpCAM were higher in viral hepatitis and cirrhosis groups. In the etiology of liver cirrhosis and HCC, CD133/EpCAM-expressing cells emerged due to either chronic HCV or HBV infection	[25]
HBV	Hepatocellular carcinoma (HCC)	HBx promotes gene expression alterations that indicate CSCs, which at least in part lead to hepatocarcinogenesis	[56]
HBV	HCC	Through PI3K/Akt signaling, miR-124 overexpression or lncRNA-MALAT1 silencing prevented HBx-induced CSC generation, stemness-related factor activation, and tumorigenicity	[58]
HCV	HCC	Through the induction of DCAMKL-1 and hepatic progenitor and stem cell-related factors, chronic HCV infection appears to predispose cells towards acquiring CSC-like traits	[60]
HCV	HCC	Treatment with stattic plus sorafenib showed a more robust impact on HCV-associated CSC mortality	[24]
HCV	HCC	HCV infection causes the CSC state by activating AKT in hepatocytes via PAI-1	[62]
EBV	Gastric carcinoma	Cancer cells that were CD44v6/v9+/+ had a higher concentration of CSC characteristics, indicating that CD44v6/v9 is a specific CSC marker for EBVaGC. The LMP2A-NFB route was used to activate the capacity to form spheres, and EBVaGC might target this pathway as a potential therapeutic target	[65]
EBV	Nasopharyngeal carcinoma	EBV, LMP1, which is linked to human cancers, including nasopharyngeal carcinoma (NPC), promotes tumor cell invasion, metastasis, and EMT. LMP1 allows NPC to develop CSC-like traits by turning on the mTORC1 and mTORC2 pathways	[64]
HCMV	Glioblastoma (GBM)	The phenotypic flexibility that HCMV infection causes in GBM cells to boost GCSC characteristics may increase the tumor’s aggressiveness	[68]
HCMV	Breast cancer	HCMV + TAMs promote proliferation, invasion, colony formation, and BCSC characteristics in inflammatory breast cancer cells via promoting the phosphorylation of p-STAT3, p-AMPK, p-PRAS40, and p-SAPK/JNK intracellular signaling molecules	[71]
ADV	GBM	NEAT1 knockdown reduced the stemness of ADV-induced GSCs. Finally, HMGB1, a DAMP that activates TLR signaling, increased the expression of stemness markers in glioma cells	[77]

## Oncolytic virotherapy

**Fig. 5:**
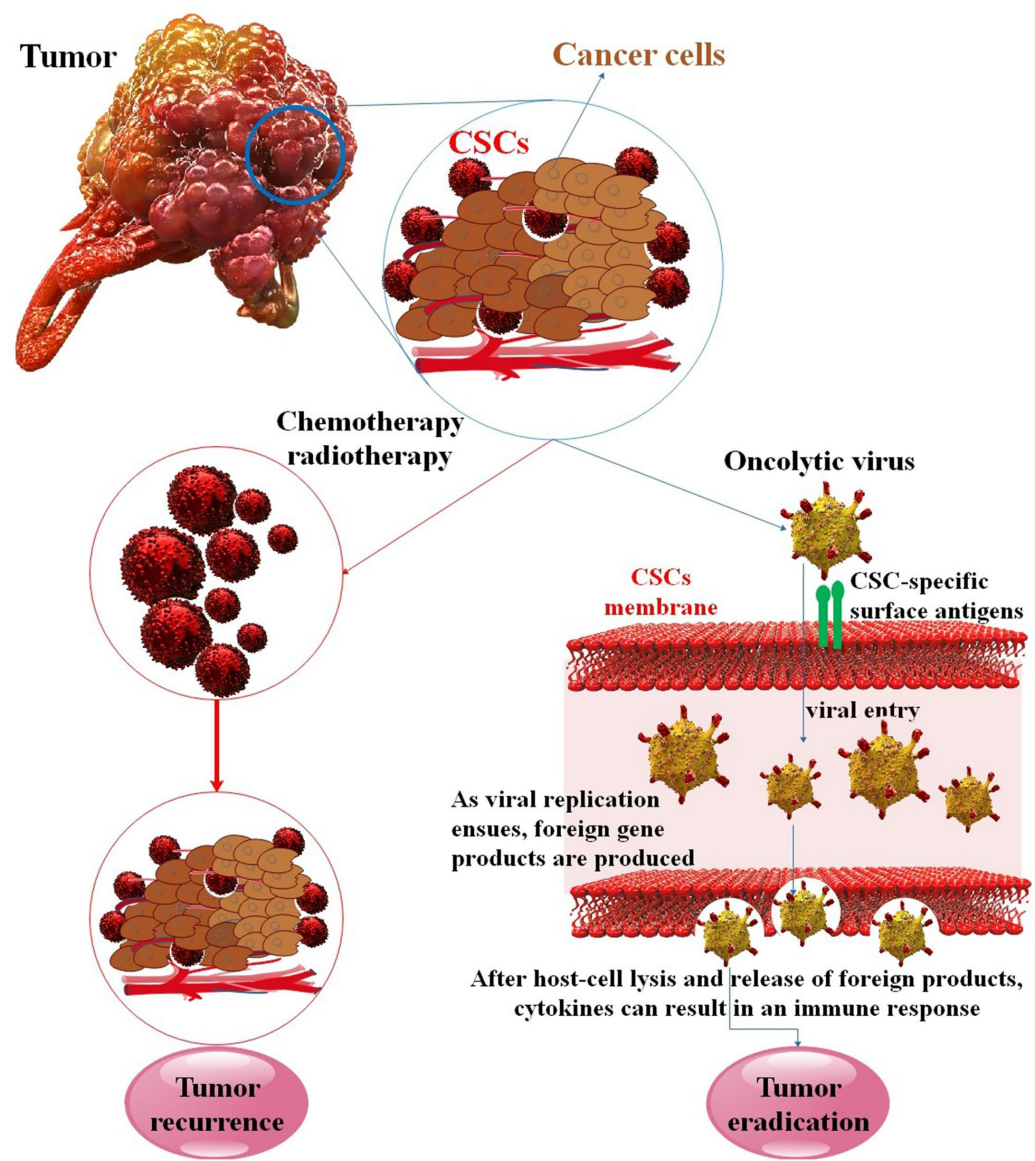
Viral oncolytic treatment in CSCs. Viruses can be delivered to the tumor site through direct injection or systemic administration. Viral alterations, such as virulence gene deletion or nonhuman host range, render normal cells non-permissive to viral infection. However, it has been observed that CSCs may be susceptible to viral infection. To facilitate viral entry, surface antigens specific to CSCs may be selected. During the progression of viral replication, foreign gene products such as cytokines (e.g., IL-12), enzymes (e.g., chondroitin), and other proteins (e.g., angiostatin) are synthesized. The release of foreign products following host-cell lysis may activate an immune response towards cancer stem cell (CSC) antigens by cytokines, leading to the activation of T cells, NK cells, and macrophages (MΦ) in uninfected cells. Enzymes or inhibitory proteins have the potential to disrupt the microenvironment of the CSC. Viruses can propagate to adjacent tumor cells [81]

OVs use biochemical distinctions between normal and tumor cells to target cancer cells while sparing normal cells preferentially. As a result, compared to standard anti-cancer treatment, oncolytic virotherapy is more selective in targeting cancer cells. Aside from a lack of specificity, conventional anti-cancer therapies frequently result in cancer relapse and incomplete cure. One explanation for this phenomenon is that some cancer cells, known as CSCs, resist standard treatments because of their capacity for self-renewal and differentiation. Researchers have been attempting to explain why OVs are best equipped to eradicate CSCs since the discovery of CSCs. It has been suggested that OVs are excellent candidates for cancer treatment for two reasons: First, the processes that lead to resistance to chemotherapy and radiation do not apply to OVs. Secondly, viruses may produce therapeutic transgenes that mainly target CSC-specific traits or CSC-dependent characteristics for self-renewal and differentiation. Preliminary research indicates that OVs may be able to successfully target CSCs in a variety of tumor types [78]. In a small number of instances, viruses may create cytotoxic proteins during cell replication, and the proteins themselves can harm cells. For example, ADVs produce the cell-cytotoxic E3 11.6-kDa death protein [79]. Generally speaking, all oncoviruses exploit metabolic pathways to induce carcinogenesis. The genetic makeup of oncoviruses, including DNA, RNA, and retroviruses, affects how they cause cancer differently. The oncoviruses that are most significant include EBV, HBV, HCV, mastadenovirus, aviadenovirus, rhadinovirus, varicellovirus, polyomavirus, ADVs, orthopoxvirus, leporipoxvirus, parvovirus, HIV, HPVs, human herpesvirus 8, and simplex virus. These viruses promote uncontrolled cell growth by interfering with the tumor p53 gene. The two oncogenes, E6 and E7, can functionally deactivate cellular genes or direct them towards degradation [31]. When cancer cells exhibit high numbers of specific viral receptors, such as CAR, CD46, or CD155, OVs have the potential to infect, multiply, and destroy the cancer cells. Increasing evidence suggests that OVs may have a significant impact on the disruption of cellular autophagy within the context of autophagy. On the other hand, OVs may be able to target CSCs because of their elevated autophagy. Infection, replication, and cell lysis have all been hypothesized as potential inducers or inhibitors of autophagy for oncolytic adenoviral treatment [80] (Fig. 5).

### Herpesvirus

Gliomas are dangerous, difficult-to-resect cerebral tumors with significant mortality and recurrence rates. IDH1 wild-type GBMs have a worse prognosis than WHO grade 4 IDH-mutant astrocytomas, according to WHO grading standards. GBM has not yet been successfully treated with any approved treatment approaches. Clinical studies have indicated that Herpes simplex virus (HSV)-1 is the most secure and efficient oncolytic virus for treating GBM. However, the molecular mechanism underlying its anticancer properties remains unclear. The oncolytic Herpes virus (oHSV)-1 was generated by eliminating the 34.5 and ICP47 genes from a particular strain of HSV-1. This genetic modification resulted in a reduction of the glioma cells’ vitality, motility, and invasiveness, as well as the suppression of microvilli formation. The transcription factor Sp1’s expression was shown to be suppressed by the infected cell polypeptide 4 (ICP4) produced by oHSV-1, which in turn decreased the expression of host invasion-related genes. The study found that oHSV-1 exhibited significant anticancer effects in vivo by suppressing the expression of Sp1 and invasion-associated genes, which are highly expressed in high-grade glioma tissue specimens. The findings indicate that Sp1 could potentially serve as a molecular indicator for the antineoplastic properties of oHSV-1 in managing glioma. Additionally, oHSV-1 impedes host cell invasion by suppressing Sp1 expression via the ICP4 pathway [82]. Treatment with HSVG47Δ deregulates the expression of non-coding RNA in GBM-CSC tumor microenvironments [83]. Researchers have shown that oncolysis caused by the oHSV selectively kills CSCs. Researchers have created cancer stem cell cultures from human GBM specimens to test the hypothesis that oHSV vectors effectively eliminate CSCs produced from human GBM-SCs. This variation in virus potency was connected to the rates of viral replication in the culture. Importantly, secondary tumorsphere formation from remaining cells was hampered by oncolytic HSV infection, suggesting that this virus can prevent cell self-renewal. Finally, an orthotopic mouse model of highly invasive GBM-SC was tested, and the results showed that intertumoral injection of G47d significantly increased animal survival. These findings demonstrated that oHSV can exert vigorous cytotoxic activity against human GBM-SCs, which should be considered for upcoming clinical trials using the vectors [84]. The cystine-glutamate antiporter xCT (SLC7A11) is a potential target for immunotherapy in breast cancer. This protein is overexpressed in mammary CSCs and plays a crucial role in maintaining their redox balance, self-renewal, and resistance to chemotherapy. Researchers utilized the bovine herpesvirus 4 (BoHV-4) vector, which has been previously demonstrated to be a secure vaccine capable of transducing cells in vivo and conferring immunogenicity to tumor antigens, to create an anti-xCT viral vaccine. In preventative and therapeutic contexts, lung metastases caused by syngeneic mammary CSCs were inhibited by immunizing BALB/c mice with BoHV-4 expressing xCT (BoHV-4-mxCT). Immunization caused T lymphocyte activation and anti-xCT antibody formation, which may directly harm CSC phenotype, self-renewal, and redox balance and facilitate antibody-dependent cell cytotoxicity (ADCC). This research opens the door to the possible use of xCT-targeting BoHV-4-based vectors in the treatment of metastatic breast cancer in the future [85]. Another study discovered a novel method to target kill LCSCs by combining the HSV-TK suicide gene, nuclide 131I irradiation, and magnetic fluid hyperthermia (MFH) with iron-platinum nanoparticles (FePt-NPs) as a carrier and CD133 antigen as a target. In comparison to any of the individual treatments, the combination intervention of pHRE-Egr1-HSV-TK/anti-CD133McAb-131I/MFH mediated by PEI-FePt-NPs may dramatically reduce LCSCs’ growth and trigger cell death in vitro [86].

### Vaccinia virus

Oncolytic vaccinia virus (VACV) therapy is being tested in clinical studies and is an appealing anti-tumor strategy. The objective of this study was to demonstrate the cytotoxic effects of VACV on chemotherapy and radiation-resistant CSCs. The discernment of CSCs from the human breast cancer cell line GI-101 A was facilitated by the augmentation of aldehyde dehydrogenase 1 (ALDH1) activity. The oncolytic VACV GLV-1h68 strain exhibited a higher proliferation rate in cells with elevated ALDH1 activity and stem cell-like properties, as opposed to cells with lower ALDH1 activity. GLV-1h68 selectively colonized and subsequently, eradicated xenograft tumors from cells exhibiting higher ALDH1 activity. Furthermore, GLV-1h68 exhibited selective proliferation in xenograft tumors originating from cells that were more tumorigenic than CD44(+)CD24(−)ESA(+) cells. Additionally, GLV-1h68 demonstrated preferential replication in CD44(+)CD24(+)ESA(+) cells derived from GI-101 A following an EMT induction. GLV-1h68 can potentially become a viable treatment option for primary and metastatic tumors, specifically those that contain cancer stem-like cells that exhibit resistance to chemotherapy and/or radiation therapy and may be responsible for tumor relapse [87]. Due to their resistance to chemotherapy and radiation therapy, stem cell-like colon cancer cells (SCCs) provide a significant therapeutic challenge for colon cancer. Researchers created an oncolytic vaccinia virus (CVV) that promoted cancer and studied how effective it was at destroying SCCs as a biotherapeutic. An evolved form of the vaccinia virus (EVV) known as CVV lacks the viral thymidine kinase (TK). Different cytotoxic pathways and cancer-favoring properties successfully overcame widespread drug resistance, killing colon cancer cells regardless of the presence of SCCs. Compared to 5-Fu-treated models, subcutaneously injected HT29 spheres grew less rapidly in CVV-treated models. CT26 spheres that were intraperitoneally administered caused tumor masses in the abdomen. Compared to 5-Fu-treated groups, CVV-treated groups demonstrated higher survival rates and decreased tumor mass formation. Fascinatingly, the combination of CVV and 5-Fu therapy increased survival rates and eliminated tumor bulk. Thus, the CVV created in this study successfully inhibits SCCs, which can be enhanced synergistically by concurrent 5-Fu therapy [88]. Glioma cell lines were infected and destroyed by recombinant Vesicular Stomatitis Virus (VSV-M51) and doubly deleted Vaccinia Virus (vvDD) in vitro, and their lifespan was extended in animal glioma models. The oncolytic capabilities of VSV-M51 and vvDD in vitro differentiated cells from fresh brain tumor tissues and generated human brain tumor stem cells (BTSCs) were further examined as a suggested ex vivo test. The grown BTSC spheres exhibited every feature of stem cells. Temozolomide (TMZ) resistant BTSCs may contract the GFP-labeled VSV-M51 and vvDD and experience cytopathic consequences. The self-renewal activity of TMZ-resistant BTSCs was reduced by the VSV-M51 and vvDD. Additionally, differentiated BTSCs were also infected by the VSV-M51 and vvDD, which had cytopathic effects [89].

### Newcastle disease virus

The oncolytic properties of the Newcastle disease virus (NDV) are known to induce oncolysis in cancer cells, along with immunogenic cell death, apoptosis, and autophagy. The treatment of NDV can potentially target a diverse range of cancer cells, such as melanoma, prostate, lung, thyroid, glioma stem cells, and GBM cells. The elimination of CSCs can be achieved with high efficiency through oncolytic NDV alone or via recombination with viral proteins [90]. The study indicates that the replication of recombinant NDV (rNDV), BC-KLQL-GFP, stimulated by prostate-specific antigen (PSA) in prostaspheres, resulted in multicycle replication that effectively induced cytolysis in prostate CSCs. The approach employed resulted in significant cytotoxicity of the CSCs derived from DU145 prostatic spheres. The efficacy of PSA-activated rNDV in vivo was demonstrated by successfully eliminating cells and prostaspheres derived from primary xenografts in an ex vivo setting. Despite being marginally more remarkable than the requisite value for the parental cell line, the EC50 (0.1 MOI) for the lysis of tumor-initiating cells remained within the therapeutic range for safety and effectiveness [91]. The study found that NDV induced cell death in glioma cells dose-dependently. Additionally, NDV was observed to decrease apoptosis and hinder self-renewal in GSCs. In this study, glioma cells and GSCs were subjected to co-culture with MSCs infected with NDV and derived from bone marrow, adipose, and umbilical cord sources. The level of apoptosis resulting from direct infection with comparable virus titers was compared to that induced by the condition media of NDV-infected MSCs. Results showed that tumor cells exhibited a higher degree of apoptosis. The results suggest that an element or agent discharged by the infected MSCs rendered glioma cells more vulnerable to the cytotoxic consequences of NDV. The study revealed that TRAIL functioned as an intermediary in the cytotoxic impact of the infected MSCs. Additionally, the research demonstrated that the combined action of TRAIL and NDV induced cell death in GSCs and glioma cells. Furthermore, the conditioned medium of the infected mesenchymal stem cells heightened the sensitivity of GBM-SCs to gamma radiation [92].

### Measles virus

MV-141.7 and MV-AC133 are two oncolytic measles viruses (MV) that specifically target CD133 and kill CD133+ tumor cells [93]. A potential CSC marker is CD133 (prominin-1). A possible method to precisely eradicate tumor cells that are CD133-positive is the use of the measles virus with CD133-targeting (MV-CD133). Through the use of an engineered MV hemagglutinin (H), selectivity was introduced at the cell entry level. The H protein revealed a CD133-specific single-chain antibody fragment (scFv) as its targeting domain and was rendered blind to its native receptors. Interestingly, while being very selective for its target cells, MV-CD133 was more effective at eliminating CD133-positive tumors than the unaltered MV-NSe. Researchers in this area have been pursuing arming technologies, receptor extension, and chimeras between MV-CD133 and vesicular stomatitis virus (VSV) to increase the antitumoral activity of MV-CD33 further. All recently developed viruses, including VSV-CD133, eliminated CD133-positive cells with extreme precision. Additionally, CD133-negative cells that were positive for the MV receptors were also killed by MV-CD46/CD133. The two antigens that encode the super cytosine deaminase, MV-CD46/CD133 and MVSCD-CD133, performed best in an orthotopic glioma model. Notably, in this tumor model, VSV-CD133 induced lethal neurotoxicity. It is possible to rule out CD133 use as a receptor as a likely cause. When administered intravenously, VSV-CD133 demonstrated the most effective oncolytic action in a subcutaneous tumor model of HCC and also considerably increased the longevity of the animals. Within the same time frame, VSV-CD133 infected a tumor area that was more than 104 times larger than that infected by MV-CD133 [94].

### Reovirus

Reovirus is a double-stranded RNA virus that enters cells through junctional adhesion molecule A (JAM-A), kills cancerous cells by both apoptotic and non-apoptotic processes, and may replicate and cause oncolysis when RAS signaling is active [95]. Oncolytic reovirus may induce tumor regression in breast cancer patients. Following reovirus treatment, the tumor’s CSC population declined at a rate comparable to that of non-CSCs. All cell types were found to have similar levels of RAS, the protein shown to mediate reovirus oncolysis, which aligns with their similar sensitivity to the virus [96]. RAS levels were comparable in the CSCs and the other tumor cells, which indicates that reovirus may successfully target CSCs that are resistant to radiation and chemotherapy. To discover if reovirus may successfully target CSCs from pediatric tumors, further research is required [81]. The direct capacity of reovirus infection to lyse the tumor cells and the induction of a robust host immune response is used to destroy tumor cells and decrease the likelihood of recurrence effectively. The effectiveness of oncolytic reovirus and safety profiles may thus be enhanced by using bioengineered stem cells as innovative carriers [30].

### Myxoma virus

Myxoma virus (MYXV) and other oncolytic poxviruses have great promise as novel weapons against human cancer. It is well known that poxviruses, such as MYXV, may attach to and begin entrance into most mammalian cells. But they then distinguish between permissive and nonpermissive cells thanks to the infected cell’s cell signaling circuitry [97]. Zemp et al. looked at the therapeutic effectiveness of the oncolytic MYXV alone or in combination with rapamycin against human brain tumor-initiating cells (BTIC) in line with these results. In cellular and animal experiments, they demonstrated that MYXV destroyed BTIC and increased the survival of BTIC-bearing mice. Furthermore, even in mice with “advanced” BTIC tumors, the combination of MYXV and rapamycin significantly improved MYXV’s antitumor activities [98]. Akt signaling is essential for cell growth, proliferation, and survival. More recently, the Akt pathway has been linked to controlling CSC survival after radiation. Furthermore, it has been demonstrated that inhibiting Akt kills brain CSCs more frequently than other brain tumor cells and lessens tumor invasiveness. These findings imply that MYXV could be a strong contender to eliminate CSCs. Few studies have examined the MYXV sensitivity of pediatric tumors or CSCs yet. In mice, a single intratumoral injection of MYXV produced a complete response in a significant portion of the aggressive pediatric cancer known as rhabdoid tumors. Early research in neuroblastoma indicates that CSCs may be vulnerable to MYXV infection. Last but not least, normal hematopoietic stem and progenitor cells were unaffected by MYXV, but adult human acute myeloid leukemia stem and progenitor cells were vulnerable to the virus’s ability to destroy them. Based on these encouraging discoveries, more analysis of MYXV in pediatric malignancies and CSCs is required [81, 97, 99, 100].

### Adenovirus

Researchers created the Ad.wnt-E1A(24 bp)-TSLC1 dual-regulated oncolytic ADV to target the Wnt and Rb signaling pathways, respectively, and to carry the TSLC1 tumor suppressor gene. Ad.wnt-E1A(24 bp)-TSLC1 is a powerful LCSC-eradicator. Effectively, Ad-wnt-E1A(24 bp)-TSLC1 could cause autophagic death. Ad-wnt-E1A(24 bp)-TSLC1 was also shown to successfully suppress the formation of transplanted tumors of hCSCs and increase mouse survival time in further animal investigations. Metastasis of hepatic CSC-like cells was also successfully inhibited by recombinant ADV-induced apoptosis [101]. In cells with high expression of human telomerase (hTERT), a ribonucleoprotein involved in cell senescence and immortalization, limitless proliferation is reported. Most normal cells have a seemingly dormant hTERT promoter, but most tumor cells have a very active one. Because of its efficacy and specificity, the hTERT promoter is a potent target for oncolytic ADVs in tumor cells. Together with three control viruses, Ad-Apoptin (Ad-VP3), Ad-hTERTp-E1A (Ad-T), and Ad-Mock, a dual cancer-specific oncolytic ADV called Ad-Apoptin-hTERTp-E1A (Ad-VT) were constructed with the Apoptin and hTERT promoter. The modified cancer-specific oncolytic ADV Ad-VT may replicate in tumor cells and induce cell death since apoptin is a tumor-specific killing protein, and the hTERT promoter precisely stimulates the viral replication required gene E1a. In research, CD44+CD24 BCSCs with significant tumorigenic potential were found using serum-free suspension culture thanks to their elevated expression of tumor stem cell markers such ALDH1A1, C-Myc, OCT4, NANOG, KLF 4, and SOX2. The particular capacity of Ad-VT to eradicate breast CSCs. Recombinant ADV Ad-VT had a killing impact on BCSCs, and the researchers effectively extracted and grew breast CSCs and discovered that the killing effect on BCSCs mainly was brought on by apoptosis [102]. In a different investigation, scientists looked at using the genetically modified oncolytic ADV OBP-301 to activate the cell cycle and eradicate dormant CSCs. GC cells with CD133 positivity exhibited stemness. OBP-301 effectively eliminated chemoradiotherapy-resistant CD133+ cancer stem-like cells. By mobilizing cell-cycle-related proteins, OBP-301 caused quiescent CD133+ cancer stem-like cells to undergo cell-cycle mobilization from G0–G1 to S/G2/M phases and subsequently die. Visualization of both proliferating CD133 non-cancer stem-like cells and dormant CD133+ cancer stem-like cells was made possible by FUCCI. The cell-cycle behavior in tumorspheres was visualized in three dimensions, and it was shown that CD133+ cancer stem-like cells maintained their stemness by staying in the G0–G1 phase. In tumorspheres and xenografts, investigators demonstrated that OBP-301 mobilized dormant cancer stem-like cells into the S/G2/M phases, where they lost viability and cancer stem-like cell characteristics and became chemosensitive [103]. Additionally, ovarian CSCs have been identified as CD133+ ovarian cancer cells. Using the Cre/LoxP switching system, the AD-CD133-Cre, which expresses Cre recombinase under the control of the CD133 promoter, and the Ad-CMV-LoxP-Neo-LoxP-tBid, which expresses tBid under the control of the CMV promoter, were effectively created. When CD133+ ovarian CSCs were co-infected with Ad-CMV-LoxP-Neo-LoxP-tBid and Ad-CD133-Cre, tBid overexpression was selectively promoted, inhibiting cell proliferation and inducing cell death. In CD133+ ovarian CSCs, the Cre/LoxP system-mediated tBid overexpression triggered the pro-apoptotic signaling pathway and increased the cytotoxicity of cisplatin [104]. A Golgi glycoprotein known as GOLPH2 (also known as GP73) is a unique tumor marker that is elevated in a variety of malignancies, including prostate cancer (PCa). The new oncolytic ADV GD55, which is GOLPH2-regulated, has a potent killing impact on hepatoma cells. The anticancer activity of GD55 on prostate cancer stem cell (CSC)-like cells are examined in a study both in vitro and in vivo. Intratumoral injection of GD55 significantly reduced the development rate of xenograft tumors in a mouse prostate CSC-like model and increased necrosis and cell death inside the tumor tissues. The findings of this investigation showed that GD55 infection had substantial anticancer effects on prostate CSC-like cells both in vitro and in vivo and may be employed in the treatment of PCa [105] (Table 2).

**Table 2:** Oncolytic viruses (OVs) inhibit CSCs in cancer treatment

Oncolytic viruses (OVs)	Cancer type	Therapeutic effects	Ref.
oHSV-1	GBM	Treatment with HSVG47Δ deregulates the expression of non-coding RNA in GBM-CSC tumor microenvironments	[83]
BoHV-4	Breast cancer	In both preventative and therapeutic scenarios, immunization of BALB/c mice with BoHV-4 expressing xCT (BoHV-4-mxCT) reduced lung metastases brought on by syngeneic mammary CSCs. Anti-xCT antibodies produced from vaccination may mediate ADCC and negatively affect CSC phenotype, self-renewal, and redox homeostasis	[85]
VACV GLV-1h68	Breast cancer	In contrast to cells with lesser ALDH1 activity, the oncolytic VACV GLV-1h68 strain multiplied more effectively in cells with greater ALDH1 activity and stem cell-like characteristics. GLV-1h68 may prove to be an effective treatment for both primary and metastatic tumors, particularly those that include cancer stem-like cells that are resistant to chemotherapy and/or radiation treatment and may be the cause of tumor recurrence	[87]
Oncolytic vaccinia virus	Colon cancer	By efficiently suppressing SCCs, the CVV created in this research can be synergistically improved by concurrent 5-Fu therapy	[88]
Vesicular stomatitis virus (VSV-ΔM51) and double deleted vaccinia virus (vvDD)	GBM	Differentiated BTSCs were additionally infected by the VSV-M51 and vvDD, which led to cytopathic consequences	[89]
NDV	GBM	A wide variety of cancer cells, including melanoma, prostate, lung, thyroid, glioma stem cells, and GBM cells might be targeted by DV treatment. The CSCs may be killed successfully with either oncolytic NDV alone or recombination with viral proteins	[90]
NDV	GBM	It was shown that TRAIL worked in concert with NDV to induce cell death in glioma cells and GSCs and that TRAIL was a mediator of the cytotoxic effects of the infected MSCs	[92]
Reovirus	Breast cancer	Patients with breast cancer may see tumor regression thanks to oncolytic reovirus. The CSC population inside the tumor decreased after reovirus therapy at the same pace as non-CSCs. RAS was discovered to be present in all cell types at comparable levels, consistent with their equal susceptibility to reovirus, and has been proven to mediate reovirus oncolysis	[96]
Adenovirus (ADV)	Hepatocellular carcinoma	Ad-wnt-E1A(△24 bp)-TSLC1 might successfully slow the development of mice’s transplanted tumors of hCSCs and increase their lifespan	[101]
ADV	Prostate cancer	GD55 infection can potentially be employed in the treatment of PCa since it exhibits powerful anticancer effects on prostate CSC-like cells in vitro and in vivo	[105]

## Genetic engineering of oncolytic viruses

Thanks to recent developments in molecular biology, researchers in the OV sector now can modify viral DNA sequences and design new viruses with enhanced specificity for cancer cells. The expression of modified receptors for cellular entry, the restriction of critical viral protein expression via cancer-specific promoters, and the deletion of viral proteins that prevent apoptosis in healthy cells are all methods currently being used in clinical trials and preclinical development involving such viruses [106]. Lytic viruses are very effective in destroying cancer cells. The selective activity of OVs may be enhanced in a variety of ways. Innate oncolytic selectivity of early OVs was linked to distinct patterns of gene and protein expression in malignant cells. However, because of the need for greater specificity, numerous strategies have been implemented to significantly boost the direct tumor specificity of OVs. Maintaining OV proliferation and downregulating proapoptotic pathways often requires deleting or modulating viral virulence factors [107]. The production of tumor-selective OVs with a tolerable safety profile and activity against a wide variety of malignancies has been made possible by recent advances in genetic engineering. By using genetic engineering and rational design, OVs may be customized to each patient’s tumor type and driver mutations. Approximately two-thirds of the OV clinical studies that have been reported have used OVs that have been genetically modified [108]. For instance, individuals with brain tumors are routinely treated in a clinical phase 2 study with pembrolizumab, an immune checkpoint inhibitor, and DNX-2401, a genetically engineered oncolytic ADV. DNX-2401 will be given intratumorally at a dosage of 1.0 mL into the brain tumor as part of this trial. Patients will have intravenous pembrolizumab, 200 mg every 3 weeks for 105 weeks (2 years), after 7 to 9 days (NCT02798406) [98, 109]. To encourage selective replication in tumor cells and reduce viral toxicity, the most common change is deleting non-essential viral genes. A multitude of preclinical and early-phase clinical studies are examining OVs that have been manipulated to generate a desired immunological response, in light of growing evidence that OVs may play an immunomodulatory function. GM-CSF is the most often added immunomodulating transgene, aiding in the stimulation of host immune responses. Many additional transgenic modifications, such ICAM-1, are similarly intended to strengthen the immune system [108, 110, 111].

ADVs have been modified to employ targeted delivery of suicide genes with highly active promoters, in addition to limiting viral replication to tumor cells. The HSV-1 TK suicide gene, which is regulated by an osteocalcin promoter, is one instance. Patients with bone metastases have elevated levels of osteocalcin. This alteration enhances the vulnerability of cancer cells with hyperactive osteocalcin promoters and limits the toxicity of the suicide gene to cells with an active osteocalcin promoter [112]. By confining the impact of the viruses to certain regions, this technique of targeting cancer cells with promoters that are tissue-specific or concentrated in the tumor may increase the therapeutic ratio by limiting adverse effects spatially [106]. Conditionally reproducing HSV-1 that has been genetically altered shows promise as a cancer treatment. They have direct cytocidal effects, may disseminate, and can reproduce in situ while exhibiting oncolytic activity. Furthermore, foreign genes may be transferred and expressed in host cells by oncolytic HSV-1. The safe administration of oncolytic HSV-1 into human brains has been shown by the phase I clinical investigation with G207, a double-mutated HSV-1, in patients with recurrent malignant gliomas. The degree of both intratumoral viral multiplication and the development of host antitumor immune responses determines the therapeutic advantages of oncolytic HSV-1. By improving these characteristics while maintaining the safety qualities, researchers create new-generation oncolytic HSV-1. G207 was transformed into G47∆ by adding a new genetic mutation. G47∆ demonstrated superiority over G207 in the following areas: (1) enhanced stimulation of human antitumor immune cells; (2) improved growth properties resulting in higher virus yields and increased cytopathic effect in vitro; (3) superior antitumor efficacy in animals with and without immunocompetence; and (4) preservation of safety in the brain of HSV-1-sensitive mice. A clinical study using G47TM in patients with progressing GBM is currently being planned. G47∆ is also a good choice for expressing foreign compounds as a backbone vector. Two DNA recombinases and a bacterial artificial chromosome have been used by researchers to build an “armed” oncolytic HSV-1 generation system that enables the precise and quick insertion of transgene(s) into the G47∆ genome. Researchers discovered that G47∆’s anticancer efficaciousness may be markedly increased by the production of immunostimulatory molecules. Based on these developments, researchers believe that oncolytic HSV-1-based oncolytic viral therapy will soon become a significant cancer therapeutic option [113, 114]. In syngeneic immunocompetent mice, Cheema et al. established a mouse glioblastoma stem cell (GSC) model that replicated tumor heterogeneity, invasiveness, vascularity, and immunosuppressive microenvironment. They investigated a genetically modified oncolytic HSV equipped with the immunomodulatory cytokine interleukin 12 (G47-mIL12) using this animal. In addition to targeting GSCs, G47-mIL12 also increases IFN-γ release, inhibits angiogenesis, and decreases the number of regulatory T cells in the tumor, indicating that G47Δ-mIL12 offers a comprehensive strategy for targeting the immune system, the tumor microenvironment, and GSCs, with potential therapeutic benefits in a rigorous GBM model [115].

Gene-viral treatment is a technique that involves introducing therapeutic genes into the genome of altered oncolytic ADVs to increase the effectiveness of the virus. Gene-viral therapy combines the benefits of both gene therapy and virotherapy, with the ability to target cancer cells and assault their angiogenesis, tumor microenvironment, and cell death signals in addition to directly killing cancer cells by oncolysis. The anti-cancer efficacy of the resulting ZD55-gene(s) has been investigated by Liu and his group after cloning multiple individual genes, including sFlt1, tumor necrosis factor-related apoptosis-inducing ligand (TRAIL), IL-24, a second mitochondria-derived activator of caspases (Smac), and others, into an oncolytic Ads. All of these ZD55-gene vectors have much stronger anti-tumor effects than either gene therapy or virotherapy by itself. They went on to investigate their plan, which included using two anti-tumor genes in gene-viral therapy in addition to two targeted promoters. Utilizing several tactics has a far greater anti-tumor impact than utilizing a single gene [116, 117]. Gene-viral treatment has also been used to eradicate CSCs. A telomerase-specific oncolytic ADV vector including the genes E1A and TRAIL was created by Zhang et al. This advertisement targeted radioresistant esophageal CSC-like cells more favorably [118].

## Future landscape

Immunotherapy is approaching a golden age due to the recent clinical triumphs of checkpoint-blocking antibodies and the identification of cancer neoantigens. The hunt for stronger tumor antigens and vaccine formulations would increase if therapeutic cancer vaccines did not yet provide the anticipated clinical effects. The fact that CSCs are the primary cause of tumor recurrence further emphasizes the potential for creating vaccinations against CSC-expressed antigens to treat metastatic cancer [85]. A more precise conceptual and practical framework of CSCs, according to experts at the 2011 Working Conference on CSCs, is crucial for their abolition [119]. The reported ineffectiveness of current therapy drives the demand for novel cancer treatments. Therefore, to fill that gap, the design of the new alternative therapies should critically address the shortcomings of conventional treatments. The inability of traditional medicines to effectively destroy the tumor’s stem cell population or CSCs, is one of the factors contributing to treatment resistance and cancer recurrence. The two critical pathways involved in how these CSCs resist treatment are drug efflux and altered cell signaling. This is similar to how Photodynamic Therapy (PDT), although being primarily regarded as an excellent alternative to invasive, fatal, and expensive medicines, has encountered the same problem where specific cancer cells are resistant to both of these methods. Fortunately, some solutions to this issue have since been offered by nanomedicine [120]. Despite the encouraging findings, the current studies have some restrictions. A bigger sample size from diverse ethnic communities is required to corroborate the results further since the sample size was minimal. To determine the therapeutic importance of the researcher’s results and to start determining if there is causation in these connections, further study is required. Anti-virus medications combined with chemotherapy might be used as a combination treatment to increase the efficacy of chemotherapy and reduce CSC resistance.

As a result, it has been discovered that most OVs tested against CSCs had more or less comparable effectiveness in killing CSCs and non-CSCs. CSCs from various cancers may not all be equally vulnerable to an oncolytic virus, however. Alternatively, it’s possible that not all OVs can affect CSCs from the same cancer. Therefore, it would be oversimplified to assume that OVs are a universal cure for all cancers. However, OVs show promise for improving cancer treatment. While several preclinical studies have suggested that OV may be effective against certain malignancies when used alone, it makes sense to combine OV with conventional therapies to maximize therapeutic benefits. One would anticipate getting an additive, if not synergistic, anti-tumor impact from combination therapy, given that OVs and traditional medicines exert their anti-tumor effect via various routes. Several studies have shown that using an oncolytic virus in conjunction with chemo or radiation treatment has a synergistic anti-tumor impact in animal models [121, 122]. The fact that CSCs share many characteristics with typical stem cells raises serious questions about employing OVs to eradicate CSCs. As a result, CSCs and regular stem cells may be similarly destroyed by OVs. However, despite the similarities between normal stem cells and CSCs, numerous studies have revealed that OVs specifically kill CSCs while sparing normal stem cells [122]. The transgene-encoding capability of OVs gives a new engineering platform to deliver immunotherapies tailored to the tumor microenvironment. OVs have shown success when used in conjunction with other cancer treatments. The development of multiarmed OVs with the ability to trigger an antitumor immune response and overcome the immunological challenges provided by CSCs and their supportive niche should be made possible by the precise selection of therapeutic transgenes [123].

## Conclusion

Given that the origin and specific properties of CSCs are still up for question, there is little doubt that these cells have shown stemness traits, including the ability to form spheres and to self-renew as well as the capability to produce tumors and to be resistant to chemo and radiation. As a result, employing various therapeutic drugs to target CSCs for antitumor therapy will be promising. Understanding how different virus types affect CSCs may aid with more effective cancer treatment and disease recurrence prevention. So, it makes sense that CSCs could resist these therapeutics. To treat human tumors, novel therapeutics based on OVs and virus-specific proteins are being developed, including adoptive cellular therapy (ACT), immunotherapy, and virotherapy. Employing an oncolytic virus as an ally in the fight against or treatment of oncogenic viruses as enemies in human malignancies is regarded as a novel therapeutic strategy. Because they are (1) overexpressed, (2) made in the wrong cell, or (3) lack negative regulatory domains, viral oncogenes constantly send out signals of proliferation. Oncogenes that are not part of the genome of the host target cell might potentially be present in viruses. Endogenous E6 and E7 genes in papillomavirus genomes, for instance, code for proteins that inhibit the tumor suppressor proteins p53 and pRb. HBV also has a gene, called PreS1, which produces an oncoprotein in human liver cells. The novel oncoprotein PreS1 is essential for the establishment and maintenance of CSCs throughout the evolution of HCC. Therefore, viral oncogenes hold a crucial part to play in CSC evolution. Viral infection and/or viral oncoproteins may play a significant role in these CSCs, however, this is not yet fully understood. New insights into viral oncogenesis and potential therapeutic techniques for treating viruses and cancer might be uncovered by using principles from cancer stem cell evolution, population genetics, thermodynamics, and systems biology. Conducting research in this field will contribute to the advancement of therapeutic approaches for cancer therapy, as well as underscore the significance of viral illness management in suppressing cancer growth and preventing disease relapse. These intriguing results pave the way for the expansion of combination and targeted anti-oncogenic medicines and may ultimately allow cutting-edge cancer therapy modalities. Future research must concentrate on approaches to use OVs to target and eliminate CSCs in light of their discovery. Next-generation viruses that target specific CSC antigens, the signaling pathways that control CSCs, and the CSC microenvironment may be created as the biology of CSCs is uncovered.

## Data Availability

Not applicable.

## References

[1] Kreso A, Dick JE (2014). Evolution of the cancer stem cell model. Cell Stem Cell.

[2] Muñoz P, Iliou MS, Esteller M (2012). Epigenetic alterations involved in cancer stem cell reprogramming. Mol Oncol..

[3] Vd P (2022). Targeting epigenetic alterations in cancer stem cells. Front Mol Med.

[4] Batlle E, Clevers H (2017). Cancer stem cells revisited. Nat Med.

[5] Ju F (2022). Characteristics of the cancer stem cell niche and therapeutic strategies. Stem Cell Res Ther.

[6] Alamgeer M (2013). Cancer stem cells in lung cancer: evidence and controversies. Respirology.

[7] Sell S, Leffert HL (2008). Liver cancer stem cells. J Clin Oncol.

[8] Gimple RC (2022). Brain cancer stem cells: resilience through adaptive plasticity and hierarchical heterogeneity. Nat Rev Cancer.

[9] Vasefifar P (2022). Nanog, as a key cancer stem cell marker in tumor progression. Gene.

[10] Bisht S (2022). Cancer stem cells: from an insight into the basics to recent advances and therapeutic targeting. Stem Cells Int.

[11] Chu M (2022). Targeting cancer stem cells by nutraceuticals for cancer therapy. Seminars in cancer biology.

[12] Baccelli I, Trumpp A (2012). The evolving concept of cancer and metastasis stem cells. J Cell Biol.

[13] Lollini P-L (2013). Preclinical vaccines against mammary carcinoma. Expert Rev Vaccines.

[14] Mullen PJ, Christofk HR (2022). The metabolic relationship between viral infection and cancer. Ann Rev Cancer Biol.

[15] Assefi M (2023). Potential use of the cholesterol transfer inhibitor U18666A as an antiviral drug for research on various viral infections. Microbial Pathog.

[16] Lin X (2023). Hepatitis E virus seroprevalence indicated a significantly increased risk selectively in patients with gastric cancer among 17 common malignancies. J Clin Med.

[17] Gholizadeh O (2023). The role of non-coding RNAs in the diagnosis of different stages (HCC, CHB, OBI) of hepatitis B infection. Microbial Pathog.

[18] Gholizadeh O (2023). Hepatitis A: viral structure, classification, life cycle, clinical symptoms, diagnosis error, and vaccination. Can J Infect Dis Med Microbiol.

[19] Gholizadeh O (2023). Review of the evidence of the effects of human papillomavirus infection and *Gardnerella vaginalis*, and their co-infection on infertility. Microbial Pathog.

[20] Ohnishi S (2013). DNA damage in inflammation-related carcinogenesis and cancer stem cells. Oxidative Med Cell Longev.

[21] Tang G, Cho M, Wang X (2022). OncoDB: an interactive online database for analysis of gene expression and viral Infection in cancer. Nucleic Acids Res.

[22] Oyouni AAA (2023). Human papillomavirus in cancer: infection, disease transmission, and progress in vaccines. J Infect Public Health.

[23] Luo Q (2023). Cancer stem cells are actually stem cells with disordered differentiation: the monophyletic origin of cancer. Stem Cell Rev Rep.

[24] Kwon Y-C (2015). Promotion of cancer stem-like cell properties in Hepatitis C virus-infected hepatocytes. J Virol.

[25] Hassan M (2023). Circulating liver cancer stem cells and their stemness-associated microRNAs as diagnostic and prognostic biomarkers for viral hepatitis-induced liver cirrhosis and hepatocellular carcinoma. Non-coding RNA Res.

[26] Smith TT (2014). Oncolytic viral therapy: targeting cancer stem cells. Oncolytic Virother.

[27] Zendedel E, Atkin SL, Sahebkar A (2019). Use of stem cells as carriers of oncolytic viruses for cancer treatment. J Cell Physiol.

[28] Huang F (2016). Oncolytic viruses against cancer stem cells: a promising approach for gastrointestinal cancer. World J Gastroenterol.

[29] Toh TB, Lim JJ, Chow EK-H (2017). Epigenetics in cancer stem. Cells Mol Cancer..

[30] Seyed-Khorrami S-M (2023). Oncolytic viruses as emerging therapy against cancers including oncovirus-induced cancers. Eur J Pharmacol.

[31] Kalarani IB, Thasneem K, Veerabathiran R (2023). Oncoviruses: future prospects of molecular mechanisms and therapeutic strategies. Oncogenic viruses.

[32] Ranjith B, Sandeep S, Veerabathiran R (2023). RNA oncoviruses and their association with cancer implications. Oncogenic viruses.

[33] Jiao X (2019). microRNA: the impact on cancer stemness and therapeutic resistance. Cells.

[34] Yilmaz V, Strati K (2019). Regulating cellular plasticity to persist: a way for tumor viruses to triumph. Curr Opin Virol.

[35] Saha A (2010). Tumor viruses and cancer biology: modulating signaling pathways for therapeutic intervention. Cancer Biol Ther.

[36] Ahmed K, Jha S (2023). Oncoviruses: how do they hijack their host and current treatment regimes. Biochim Biophys Acta (BBA) Rev Cancer.

[37] Meng X (2023). The roles of different microRNAs in the regulation of cholesterol in viral hepatitis. Cell Commun Signal.

[38] Mani SKK, Andrisani O (2018). Hepatitis B virus-associated hepatocellular carcinoma and hepatic cancer stem cells. Genes.

[39] Bahnassy AA (2014). Circulating tumor and cancer stem cells in Hepatitis C virus-associated liver disease. World J Gastroenterol.

[40] Baj J (2022). The involvement of human papilloma virus in gastrointestinal cancers. Cancers.

[41] Boulet G (2007). Human papillomavirus: E6 and E7 oncogenes. Int J Biochem Cell Biol.

[42] Barbosa MS (1991). In vitro biological activities of the E6 and E7 genes vary among human papillomaviruses of different oncogenic potential. J Virol.

[43] Labarge B (2022). Human papilloma virus integration strictly correlates with global genome instability in head and neck cancer. Mol Cancer Res.

[44] Muresu N (2022). Prevalence of human papilloma virus infection in bladder cancer: a systematic review. Diagnostics.

[45] Ortiz-Sánchez E (2016). Characterization of cervical cancer stem cell-like cells: phenotyping, stemness, and human papilloma virus co-receptor expression. Oncotarget.

[46] Mendoza–Almanza G (2019). Cervical cancer stem cells and other leading factors associated with cervical cancer development. Oncol Lett.

[47] Saraf S, Suresh P, Das RK (2023). Unravelling the role of EMT in OSCC: a quick peek into HPV-mediated pathogenesis. Oral Oncol Rep.

[48] Olivero C (2018). HPV-induced field cancerisation: transformation of adult tissue stem cell into cancer stem cell. Front Microbiol.

[49] Tang AL (2013). Head and neck cancer stem cells: the effect of HPV—an in vitro and mouse study. Otolaryngol Head Neck Surg.

[50] Reid P (2020). Influence of the human papillomavirus on the radio-responsiveness of cancer stem cells in head and neck cancers. Sci Rep.

[51] Reid P (2019). Diversity of cancer stem cells in head and neck carcinomas: the role of HPV in cancer stem cell heterogeneity, plasticity and treatment response. Radiother Oncol.

[52] Organista–Nava J (2019). Cervical cancer stem cell-associated genes: prognostic implications in cervical cancer. Oncol Lett.

[53] Liou J-W, Mani H, Yen J-H (2022). Viral Hepatitis, cholesterol metabolism, and cholesterol-lowering natural compounds. Int J Mol Sci.

[54] Rozeik MS (2017). Evaluation of CD44 and CD133 as markers of liver cancer stem cells in Egyptian patients with HCV-induced chronic liver diseases versus hepatocellular carcinoma. Electron Physician.

[55] Liu Z (2017). Hepatitis B virus PreS1 facilitates hepatocellular carcinoma development by promoting appearance and self-renewal of liver cancer stem cells. Cancer Lett.

[56] Arzumanyan A (2011). Does the Hepatitis B antigen HBx promote the appearance of liver cancer stem cells? HBx and liver CSC. Cancer Res.

[57] Zhu M (2017). HBx drives alpha fetoprotein expression to promote initiation of liver cancer stem cells through activating PI3K/AKT signal pathway. Int J Cancer.

[58] He B (2019). Interaction of lncRNA-MALAT1 and miR‐124 regulates HBx‐induced cancer stem cell properties in HepG2 through PI3K/Akt signaling. J Cell Biochem.

[59] Xiao Y (2017). The recent advances on liver cancer stem cells: biomarkers, separation, and therapy. Anal Cell Pathol.

[60] Ali N (2011). Hepatitis C virus-induced cancer stem cell-like signatures in cell culture and murine tumor xenografts. J Virol.

[61] Shirasaki T (2018). The osteopontin-CD44 axis in hepatic cancer stem cells regulates IFN signaling and HCV replication. Sci Rep.

[62] Nam D-E (2021). Elevation of plasminogen activator Inhibitor-1 promotes differentiation of cancer stem-like cell state by hepatitis C virus infection. J Virol.

[63] Kondo S (2011). Epstein–Barr virus latent membrane protein 1 induces cancer stem/progenitor-like cells in nasopharyngeal epithelial cell lines. J Virol.

[64] Zhu N (2022). Epstein–Barr virus LMP1-Activated mTORC1 and mTORC2 coordinately promote nasopharyngeal cancer stem cell properties. J Virol.

[65] Yasui M (2020). Cancer stem cells in Epstein–Barr virus‐associated gastric carcinoma. Cancer Sci.

[66] Herbein G (2018). The human cytomegalovirus, from oncomodulation to oncogenesis. Viruses.

[67] Herbein G (2022). High-risk oncogenic human cytomegalovirus. Viruses.

[68] Fornara O (2016). Cytomegalovirus Infection induces a stem cell phenotype in human primary glioblastoma cells: prognostic significance and biological impact. Cell Death Differ.

[69] Soroceanu L (2015). Cytomegalovirus immediate-early proteins promote stemness properties in glioblastoma. Cancer Res.

[70] Cobbs CS (2008). Modulation of oncogenic phenotype in human glioma cells by cytomegalovirus IE1-mediated mitogenicity. Cancer Res.

[71] Mohamed HT (2022). Inflammatory breast cancer: the secretome of HCMV+ tumor-associated macrophages enhances proliferation, invasion, colony formation, and expression of cancer stem cell markers. Front Oncol.

[72] Isaguliants M (2021). Oncogenic effects of HIV-1 proteins, mechanisms behind. Cancers.

[73] Chahroudi A, Silvestri G, Lichterfeld M (2015). T memory stem cells and HIV: a long-term relationship. Curr HIV/AIDS Rep.

[74] Finley J (2017). Elimination of cancer stem cells and reactivation of latent HIV-1 via AMPK activation: common mechanism of action linking inhibition of tumorigenesis and the potential eradication of HIV-1. Med Hypotheses.

[75] Machitani M (2011). Adenovirus vector-derived VA-RNA-mediated innate immune responses. Pharmaceutics.

[76] Hu Y-Y (2014). Hif-1α and Hif-2α differentially regulate notch signaling through competitive interaction with the intracellular domain of notch receptors in glioma stem cells. Cancer Lett.

[77] Zang J (2020). Adenovirus Infection promotes the formation of glioma stem cells from glioblastoma cells through the TLR9/NEAT1/STAT3 pathway. Cell Commun Signal.

[78] Ding J (2014). Oncolytic virus as a cancer stem cell killer: progress and challenges. Stem Cell Investig.

[79] Zou A (2004). Overexpression of adenovirus E3-11.6 K protein induces cell killing by both caspase-dependent and caspase-independent mechanisms. Virology.

[80] Nazio F (2019). Autophagy and cancer stem cells: molecular mechanisms and therapeutic applications. Cell Death Differ.

[81] Friedman GK (2012). Targeting pediatric cancer stem cells with oncolytic virotherapy. Pediatr Res.

[82] Zhang J (2023). Oncolytic HSV-1 suppresses cell invasion through downregulating Sp1 in experimental glioblastoma. Cell Signal.

[83] Vazifehmand R (2022). The evaluation expression of non-coding RNAs in response to HSV-G47∆ oncolytic virus infection in glioblastoma multiforme cancer stem cells. J Neurovirol.

[84] Martuza R, et al. Use of oncolytic herpes viruses for killing cancer stem cells. Google Patents; 2014.

[85] Donofrio G (2018). Bovine herpesvirus 4-based vector delivering the full length xCT DNA efficiently protects mice from mammary cancer metastases by targeting cancer stem cells. Oncoimmunology.

[86] Lin M (2020). A combination therapy of pHRE-Egr1-HSV-TK/Anti-CD133McAb-131I/MFH mediated by FePt nanoparticles for liver cancer stem cells. J Nanomater..

[87] Wang H (2012). Oncolytic vaccinia virus GLV-1h68 strain shows enhanced replication in human breast cancer stem-like cells in comparison to breast cancer cells. J Transl Med.

[88] Yoo SY (2016). A cancer-favoring oncolytic vaccinia virus shows enhanced suppression of stem-cell like colon cancer. Oncotarget.

[89] Jiang B (2017). Temozolomide resistant human brain tumor stem cells are susceptible to recombinant vesicular stomatitis virus and double-deleted vaccinia virus in vitro. Biomed Pharmacother.

[90] Zhang Y-N (2020). Recent advances in targeting cancer stem cells using oncolytic viruses. Biotechnol Lett.

[91] Raghunath S (2017). Genetically engineered oncolytic Newcastle disease virus mediates cytolysis of prostate cancer stem like cells. J Biotechnol.

[92] Kazimirsky G (2016). Mesenchymal stem cells enhance the oncolytic effect of Newcastle disease virus in glioma cells and glioma stem cells via the secretion of TRAIL. Stem Cell Res Ther.

[93] Liu Y-C, Yeh C-T, Lin K-H (2020). Cancer stem cell functions in hepatocellular carcinoma and comprehensive therapeutic strategies. Cells..

[94] Kleinlützum D (2017). Enhancing the oncolytic activity of CD133-targeted measles virus: receptor extension or chimerism with vesicular stomatitis virus are most effective. Front Oncol.

[95] Müller LM (2021). Reovirus-induced cell-mediated immunity for the treatment of multiple myeloma within the resistant bone marrow niche. J Immunother Cancer.

[96] Marcato P (2009). Oncolytic reovirus effectively targets breast cancer stem cells. Mol Ther.

[97] Kim M (2009). Myxoma virus targets primary human leukemic stem and progenitor cells while sparing normal hematopoietic stem and progenitor cells. Leukemia.

[98] Bahreyni A (2019). Therapeutic potency of oncolytic virotherapy-induced cancer stem cells targeting in brain tumors, current status, and perspectives. J Cell Biochem.

[99] Wu Y (2008). Oncolytic efficacy of recombinant vesicular stomatitis virus and myxoma virus in experimental models of rhabdoid tumors. Clin Cancer Res.

[100] Cripe TP (2009). Targeting cancer-initiating cells with oncolytic viruses. Mol Ther.

[101] Zhang J (2017). A novel oncolytic adenovirus targeting wnt signaling effectively inhibits cancer-stem like cell growth via metastasis, apoptosis and autophagy in HCC models. Biochem Biophys Res Commun.

[102] Li W (2021). Anti-tumour effects of a dual cancer‐specific oncolytic adenovirus on breast cancer stem cells. J Cell Mol Med.

[103] Yano S (2013). A genetically engineered oncolytic adenovirus decoys and lethally traps quiescent cancer stem-like cells in S/G2/M phases. Clin Cancer Res.

[104] Long Q (2017). Adenovirus-mediated truncated bid overexpression induced by the Cre/LoxP system promotes the cell apoptosis of CD133+ ovarian cancer stem cells. Oncol Rep.

[105] Ying C (2018). GOLPH2-regulated oncolytic adenovirus, GD55, exerts strong killing effect on human prostate cancer stem-like cells in vitro and in vivo. Acta Pharmacol Sin.

[106] Jhawar SR (2017). Oncolytic viruses—natural and genetically engineered cancer immunotherapies. Front Oncol.

[107] Tian Y, Xie D, Yang L (2022). Engineering strategies to enhance oncolytic viruses in cancer immunotherapy. Signal Transduct Target Ther.

[108] Lauer UM, Beil J (2022). Oncolytic viruses: challenges and considerations in an evolving clinical landscape. Future Oncol.

[109] Garber K (2006). China approves world’s first oncolytic virus therapy for cancer treatment. J Natl Cancer Inst.

[110] Macedo N (2020). Clinical landscape of oncolytic virus research in 2020. J Immunother Cancer.

[111] Bahreyni A (2023). A combination of genetically engineered oncolytic virus and melittin-CpG for cancer viro-chemo-immunotherapy. BMC Med.

[112] Koeneman KS (2000). Osteocalcin-directed gene therapy for prostate-cancer bone Metastasis. World J Urol.

[113] Todo T (2008). Oncolytic virus therapy using genetically engineered herpes simplex viruses. Front Biosci-Landmark.

[114] Faghihkhorasani A (2023). The potential use of bacteria and bacterial derivatives as drug delivery systems for viral infection. Virol J.

[115] Cheema TA (2013). Multifaceted oncolytic virus therapy for glioblastoma in an immunocompetent cancer stem cell model. Proc Natl Acad Sci.

[116] Liu XY (2006). Targeting gene-virotherapy of cancer and its prosperity. Cell Res.

[117] Tong Y, Qian W (2014). Targeting cancer stem cells with oncolytic virus. Stem Cell Investig.

[118] Zhang X (2008). Treatment of radioresistant stem-like esophageal cancer cells by an apoptotic gene-armed, telomerase-specific oncolytic adenovirus. Clin Cancer Res.

[119] Valent P (2012). Cancer stem cell definitions and terminology: the devil is in the details. Nat Rev Cancer.

[120] Chizenga EP, Abrahamse H (2020). Nanotechnology in modern photodynamic therapy of cancer: a review of cellular resistance patterns affecting the therapeutic response. Pharmaceutics.

[121] Adusumilli PS (2005). Radiation therapy potentiates effective oncolytic viral therapy in the treatment of lung cancer. Ann Thorac Surg.

[122] Chaurasiya S, Chen NG, Warner SG (2018). Oncolytic virotherapy versus cancer stem cells: a review of approaches and mechanisms. Cancers.

[123] Crupi MJ, Bell JC, Singaravelu R (2019). Concise review: targeting cancer stem cells and their supporting niche using oncolytic viruses. Stem Cells.

